# Pharmacogenomics of treatment toxicities in pediatric B-Cell ALL: toward safer precision therapy

**DOI:** 10.3389/fphar.2026.1806729

**Published:** 2026-05-19

**Authors:** Meriem Lameri, Tarek Kamergi, Ameni Brahim, Imen Abdallah, Nessrine Mezzi, Sarah Zerei, Alia Benkahla, Manel Chaabane, Chema Drira, Hajer Felfel, Yosr Ben Abdennebi, Miriam Razgallah Khrouf, Dorra Amor, Lilia Romdhane, Hend Chaker

**Affiliations:** 1 Centre National de Transfusion Sanguine, Tunis, Tunisia; 2 Pharmaceutical Sciences A Department, Clinical Biology A Department, Universite de Monastir, Faculte de Pharmacie de Monastir, Monastir, Tunisia; 3 Centre National de Greffe de la Moelle Osseuse, Tunis, Tunisia; 4 Pharmaceutical Sciences B Department, Universite de Monastir, Faculte de Pharmacie de Monastir, Monastir, Tunisia; 5 Research Laboratory of Chemical, Galenic and Pharmacological Development of Medicines (LR12ES09), Universite de Monastir Faculte de Pharmacie de Monastir, Monastir, Tunisia; 6 Department of Biochemistry, LR12SP11, Hopital Sahloul, Sousse, Tunisia; 7 Laboratory of Biomedical Genomics and Oncogenetics, LR 16 IPT 05, Institut Pasteur de Tunis, Tunis, Tunisia; 8 National Agency of Medicines and Health Products, Tunis, Tunisia; 9 Bioinformatics Laboratory, Biomathematics, and Biostatistics (LR16IPT09), Institut Pasteur de Tunis, Tunis, Tunisia; 10 Clinical Biology A Department, Universite de Monastir Faculte de Pharmacie de Monastir, Monastir, Tunisia; 11 Pediatric Clinical Hematology, Hopital Aziza Othmana, Tunis, Tunisia; 12 Department of Life Sciences, Faculty of Sciences of Bizerte, University of Carthage, Tunis, Tunisia; 13 Laboratory of Medical Genetics, Institut Pasteur de Tunis, Tunis, Tunisia

**Keywords:** acute lymphoblastic leukemia, drug toxicity, genotype-guided therapy, *NUDT15*, pharmacogenomics, precision medicine, *TPMT*

## Abstract

Pharmacogenomics (PGx) has emerged as a key strategy to predict and prevent drug hypersensitivity reactions and treatment-related toxicities, thereby improving therapeutic adherence and survival in cancer care. Despite major advances in survival outcomes for pediatric B-Acute Lymphoblastic Leukemia (B-ALL), treatment-related toxicities remain a significant clinical challenge. Notably, treatment de-escalation strategies for low-risk leukemia are currently being explored in several clinical trials to reduce therapy-related toxicities. This review focuses on the mechanisms of drug-induced toxicity and their association with pharmacogenetic determinants in B-ALL therapy. Among the pharmacogenetic factors influencing toxicity of commonly used B-ALL treatments, variants in the *TPMT* and *NUDT15* genes, both involved in the metabolism of 6-mercaptopurine, represent the most robust and clinically validated predictors. Emerging evidence also links additional genetic variants to toxicities associated with other key agents used in ALL treatment regimens, including variants in *SLCO1B1* associated with methotrexate-related gastrointestinal toxicity and variants in *CEP72* associated with vincristine-induced neuropathy. The integration of pharmacogenomic biomarkers into clinical protocols, enabling genotype-guided dose adjustment, may represent a valuable strategy to avoid toxicity and improve overall cancer outcomes. However, further studies and innovative approaches are required to validate emerging biomarkers and facilitate their translation into routine clinical practice.

## 1 Introduction

Pharmacogenomics (PGx) has emerged as a central component of precision oncology, as genomic variability substantially contributes to interindividual differences in treatment response and toxicity profiles. Pharmacogenetic biomarkers therefore represent a valuable tool for the individualization of anticancer therapy, guiding drug selection and dose optimization, with the aim of minimizing treatment-related toxicity while maintaining therapeutic efficacy.

Leukemia is the 13th most diagnosed cancer worldwide, with more than 487,000 new cases reported in 2022 (Sung et al., 2021). In Pediatric cancer, Leukemia is the most common cancer, and the leading cause of cancer-related mortality in children. Globally, in 2022, 77.660 new cases and 31.106 deaths from childhood leukemia were estimated, accounting for 36.79% of all incident childhood cancers and 39.66% of childhood cancer–related deaths. Among leukemia subtypes, Acute Lymphoblastic Leukemia (ALL) is the most prevalent in the pediatric population, representing approximately 66.73% of newly diagnosed leukemia cases (Sung et al., 2021; Wang et al., 2025). ALL may be of B- (B-ALL) or T-lymphoid (T-ALL) lineage, accounting, respectively, for 85% and 15% of all cases (Jędraszek et al., 2022).

Multi-agent chemotherapy is administered across the induction, consolidation, and maintenance phases in the treatment of childhood ALL, referred to as B-Cell Lymphoblastic Leukemia/Lymphoma in the fifth edition of the World Health Organization Classification of Haematolymphoid Tumours (Alaggio et al., 2022).

Several treatment protocols are used worldwide for the management of B-ALL. These include the Collaborative Treatment Protocol for Children and Adolescents with Acute Lymphoblastic (AIEOP-BFM 2017 ALL) in Europe, the Children’s Oncology Group protocols (COG-AALL series) in the United States, and the United Kingdom National Randomized Trial for ALL and Lymphoma 2011 (UKALL2011) in the United Kingdom. Although these protocols differ in risk stratification and treatment intensity, they share a common backbone of multi-agent chemotherapy aimed at maximizing cure rates while minimizing treatment-related toxicities (Jędraszek et al., 2022). These treatment regimens commonly include corticosteroids (dexamethasone and prednisone) and cytotoxic agents, mainly vincristine, L-asparaginase, methotrexate, and anthracyclines (e.g., daunorubicin or doxorubicin), administered across different treatment phases, beginning with induction. Additional agents, such as 6-mercaptopurine (6-MP) and cyclophosphamide, are incorporated during consolidation and maintenance phases to sustain remission (Jędraszek et al., 2022). Recent therapeutic advances have expanded treatment options to include targeted therapies such as tyrosine kinase inhibitors (TKIs), as well as immunotherapies, including the anti-CD19 bispecific antibody (Blinatumomab), and CAR T-cell therapy (Gökbuget et al., 2024; Jędraszek et al., 2022).

Major advances in risk-adapted therapy and therapeutic innovations in B-ALL have substantially improved patient outcomes, with the 5-year survival rate of childhood cancers now approaching 80% in high-income countries (Wu S. et al., 2022). However, these improvements have not been uniform across all regions, with rates remaining as low as 40% in low- and middle-income countries (LMICs), where over 80% of children with cancer are located (Hunleth et al., 2024).

Despite overall remarkable improvements in survival in B-Cell ALL, severe treatment-related toxicities remain a major clinical challenge, as they may lead to therapy delays and dose reductions, thereby negatively affecting overall outcomes (Franca et al., 2017).

Chemotherapy-induced toxicities remain a significant clinical concern in ALL. Common adverse effects include myelosuppression, hypersensitivity reactions, hepatotoxicity, anthracycline-induced cardiotoxicity, and neurotoxicity. These toxicities often necessitate dose reductions, treatment delays, or permanent modifications of therapeutic regimens (Al-Mahayri et al., 2021).

In a pediatric cohort treated according to the AIEOP-BFM 2000 protocol, 49.4% of patients experienced at least one severe (Grade III/IV) of gastrointestinal, hepatic, or neurological toxicity, during the induction or consolidation phases (Franca et al., 2017). Asparaginase-associated toxicity is another important concern. Clinical hypersensitivity is the most common adverse event leading to treatment discontinuation, occurring in up to 30% of patients receiving E. coli–derived asparaginase (Hijiya and van der Sluis, 2016). Asparaginase allergic reactions have decreased in recent years with the introduction of pegylated Pegaspargase (Oncaspar) instead of native *E. coli* asparaginase. However, the latter remains in use in most low- and middle-income countries. Additionally, the use of anthracyclines increases the risk of cardiotoxicity in both pediatric and adult patients with ALL (Cardinale et al., 2020; Aslam et al., 2021).

The incorporation of novel therapeutic agents—including tyrosine kinase inhibitors (TKIs), CD19-directed CAR T-cell therapy, and the bispecific T-cell engager Blinatumomab has improved outcomes for specific subtypes of ALL (ALL). TKIs have significantly improved survival in patients with Philadelphia chromosome–positive ALL and Philadelphia chromosome–like (Ph-like) B-ALL, while immunotherapies have demonstrated substantial efficacy in relapsed or refractory disease. However, these innovative therapies are also associated with distinct toxicity profiles. For instance, TKIs may induce cardiovascular toxicities (Kong et al., 2012; Vivona et al., 2012), whereas immunotherapies can lead to immune-related adverse events such as cytokine release syndrome (CRS). Management of CRS often requires targeted interventions, including interleukin-6 (IL-6) receptor blockade with Tocilizumab (Frey and Porter, 2016; Zhang et al., 2018).

Given the persistent challenge of treatment-related toxicities, contemporary clinical trials increasingly investigate therapy de-escalation strategies guided by leukemia risk stratification to reduce treatment burden while maintaining therapeutic efficacy. For example, the randomized controlled trial UKALL2003 evaluates treatment reduction in children and young adults with low-risk ALL, defined by minimal residual disease (MRD) (Vora et al., 2012). Similarly, the ALLTogether trial, a large European multicenter study including infants, children and young adults with ALL, aims to assess therapy de-escalation through refined risk stratification including different criteria, and adjustment of chemotherapy intensity in low-risk patients (Heyman, 2025).

With ongoing clinical efforts focused on treatment de-escalation and transitioning to drugs with fewer side effects, the integration of preemptive pharmacogenetic genotyping holds significant potential to reduce treatment-related toxicities and improve outcomes. This review provides an overview of key pharmacogenes associated with drug-related toxicities in B-ALL therapy, presents clinically actionable biomarkers, evaluates the strength of current evidence, examines population-specific allele frequencies, and outlines pathways and opportunities for implementation.

## 2 Methodology

We conducted a comprehensive literature review to identify genetic associations with drug-induced hypersensitivity reactions and toxicities in the treatment of acute lymphoblastic leukemia (ALL). The methodology consisted of several steps.

First, we established an exhaustive list of drugs commonly used in the induction, consolidation, and maintenance phases of ALL treatment protocols. For each drug, we performed a targeted PubMed search using combinations of the drug name with the keywords “pharmacogenetis”, “toxicity”, and “adverse reactions”. In parallel, we queried the PharmGKB database to retrieve curated pharmacogenetic annotations linked to each drug.

To ensure broader coverage, we conducted an additional PubMed search using the combined terms “pharmacogenetics” AND “acute leukemia” AND “toxicity”, with the objective of identifying review articles relevant to the topic.

For each eligible study or pharmacogenetic annotation, data extraction captured the following elements:

- Drug name and corresponding gene–variant associations.
- Type of adverse reaction or toxicity
- Proposed mechanism of drug-induced toxicity or hypersensitivity
- Sample size, odds ratio (OR), and 95% confidence interval (CI)
- Level of evidence (e.g., clinical guideline, meta-analysis, cohort or case-control study)
- Recommendations provided in clinical practice guidelines, when available

To characterize population-specific genetic variability, allele frequencies were obtained from population database, the Genome Aggregation Database (gnomAD). In addition, frequencies of HLA alleles were extracted from the Allele Frequency Net Database (http://www.allelefrequencies.net).

Finally, we searched for literature addressing pharmacoeconomic aspects and implementation challenges to contextualize the clinical utility, feasibility, and future perspectives of using genetic biomarkers as predictive tools for treatment-related toxicities.

## 3 Drug-gene associations and toxicity in ALL

### 3.1 Mercaptopurine/thiopurine

6-Mercaptopurine (6-MP) is a key component of maintenance therapy in ALL, particularly in combination with weekly methotrexate to maintain remission and reduce relapse risk (Pui et al., 2014; Schmiegelow et al., 2014). The therapeutic challenge arises from its narrow benefit–toxicity margin, where insufficient exposure increases relapse risk, while excessive exposure predisposes to severe, sometimes life-threatening myelosuppression (Relling and Evans, 2015).

#### 3.1.1 Mechanism of drug-induced toxicity

The cytotoxicity of 6-MP arises from its intracellular metabolism into 6-thioguanine nucleotides (6-TGNs), which incorporate into DNA and RNA, thereby disrupting replication and repair processes. The balance of active metabolite formation is tightly regulated by enzymes such as thiopurine S-methyltransferase (TPMT) and nudix hydrolase 15 (NUDT15). Loss-of-function variants in these genes alter thiopurine metabolism. TPMT deficiency reduces thiopurine methylation, resulting in excessive accumulation of 6-TGNs and severe myelosuppression. In contrast, NUDT15 deficiency impairs the hydrolysis of thioguanine triphosphates, promoting their incorporation into DNA and leading to early, pronounced leukopenia and, in some populations, alopecia (Moriyama et al., 2016; Yang et al., 2014).

In addition to hematological toxicity, 6-MP is associated with hepatotoxicity, which is primarily linked to the accumulation of methylated metabolites, particularly 6-methylmercaptopurine (6-MMP). Elevated 6-MMP levels have been correlated with liver enzyme abnormalities and hepatic dysfunction. While TPMT activity influences the balance between 6-TGN and 6-MMP production, emerging evidence suggests that inosine triphosphate pyrophosphatase (ITPA) may further modulate thiopurine metabolism (Szoszkiewicz et al., 2026). ITPA deficiency leads to the accumulation of non-canonical purine metabolites and has been associated with increased 6-MMP levels and a higher risk of hepatotoxicity in some clinical cohorts. However, the clinical utility of ITPA genotyping remains less well established compared to TPMT and NUDT15 and it is not currently incorporated into routine dosing guidelines (Szoszkiewicz et al., 2026). Collectively, these pharmacogenetic determinants contribute to interindividual variability in both hematologic and hepatic toxicities, underscoring the importance of genotype-guided thiopurine therapy to improve treatment safety (Franca et al., 2017; Szoszkiewicz et al., 2026).

#### 3.1.2 Genetic variants associated with thiopurine toxicity

Common non-functional *TPMT* variant alleles include TPMT*2, *3A, *3B and *3C, which together account for the majority of reduced-function alleles associated with diminished TPMT enzymatic activity. Individuals who are heterozygous for one no-function allele exhibit intermediate metabolizer phenotypes (IM) and are at increased risk of 6-mercaptopurine–induced myelosuppression at conventional doses. Homozygous carriers of two no-function alleles are classified as poor metabolizers (PM), with a high likelihood of severe or life-threatening toxicity. This underscores the clinical importance of genotype-guided dosing in thiopurine therapy (Maillard et al., 2024). The distribution of these variants demonstrates marked ethnic variability: TPMT*3A (rs1800460) is more frequently observed in European ancestry groups, whereas TPMT*3C (rs1142345) predominates among African populations (Zhou, 2006) (Table 1). In North African population, reduced-function *TPMT* alleles, including *3A and *3C have been identified as contributors to interindividual variability in thiopurine metabolism, with documented effects on enzyme activity and metabolite exposure that are mechanistically linked to differential susceptibility to thiopurine-induced hematological toxicity (Ben Salah et al., 2011; Chadli et al., 2024; Souissi et al., 2025).

**TABLE 1:** Genetic variants associated with 6-Mercaptopurine toxicity.

Toxicity phenotype	Gene	Variant (rsID/HGVS nomenclature)	Study design/Genetic approach	Sample size, OR (95% CI); p value)	References	Allele frequency in reference populations	Clinical guideline/Clinical recommendations
Myelosupression	*TPMT*	TPMT*2 NM_000367.5: c.238G>C (rs1800462)	Retrospective cohort study/candidate gene association	N = 210; p < 0.001	Moradveisi et al. (2019)	Global: 0.2% European: 0.2% African: 0.0% East Asian: 0.0%	CPIC: mandatory dose reduction; alternative therapy in poor metabolizers (PM)
		TPMT*3A NM_000367.5: c.460G>A (rs1800460)	Retrospective cohort study (meta-analysis)/candidate gene association study	N = 1768, (95% CI: −31.1 to −12.5); p = 4.5 × 10^−6^	Maillard et al., 2024	Global: 3% European: 3% African: 0.9% East Asian: 0.0%	CPIC: 30%–80% dose reduction in intermediate metabolizers (IM); avoid standard dosing in PM
		TPMT*3A NM_000367.5: c.719A>G (rs1142345)				Global: 4% European: 4% African: 5% East Asian: 1%	
Early leukopenia	*NUDT15*	NM_018283.4: c.415C>T (rs116855232)	Retrospective cohort study/candidate gene association study	N = 105, OR = 9.63 (95% CI: 2.764–33.514); p = 3.75 × 10^−4^	Zhou et al., 2018	Global: 0.3% European: 0.2% African: 0.1% East Asian: 9%	CPIC: major dose reduction; avoid standard dosing
Myelosuppression (additive effects)	*TPMT + NUDT15*	TPMT (*2, *3A, *3B, *3C) and NUDT15 (*2, *3, *9)	Retrospective cohort study/candidate gene association study	N = 1768, (95% CI: −31.1 to −12.5); p = 4.5 × 10^−6^	Maillard et al., 2024	NA	CPIC: TPMT IM and NUDT15 IM: initiate thiopurines at 20%–50% of standard doses in dual IM patients to mitigate toxicity risk. Any combination with TPMT PM and/or NUDT15 PM: initiate therapy with drastically reduced starting doses. Reduce starting dose by 10-fold and reduce frequency to thrice weekly instead of daily.
Hematological toxicity	*ITPA*	NM_033453.4: c.124+21A>C (rs7270101)	Retrospective cohort/candidate gene association study	N = 210; p < 0.001	Moradveisi et al., 2019	Global: 10% European: 12% African: 0.3% East Asian: 7%	NA
Gastrointestinal (GI) toxicity and neurological (NEU) adverse events	*ITPA*	NM_033453.4: c.94C>A (rs1127354)	Retrospective cohort study/candidate gene association study	N = 508, GI: OR = 7.73 (95% CI: 1.04–57.34); p = 0.046 NEU: OR = 13.23 (95% CI: 1.74–100.65); p = 0.013	Franca et al., 2017	European: 7.06% African: 4.46% East Asian: 16.87% South Asian:12.17%	NA

*TPMT*, Thiopurine S-méthyltransférase; *NUDT15*, Nudix hydrolase 15; CPIC, clinical pharmacogenomics implementation consortium; PM, poor metabolizer; IM:intermediate metabolizer; 6-TGN, 6-thioguanine nucleotides; *ITPA*, inosine triphosphate pyrophosphatase; N/A, Not Available. Allele frequencies were obtained from the Genome Aggregation Database (gnomAD).

For *NUDT15*, the missense variant p. Arg139Cys (rs116855232) represents a major determinant of thiopurine intolerance in Asian and Hispanic populations (Yang et al., 2014). Zhou et al. identified the *NUDT15* c.415C>T (rs116855232) variant as the most robust predictor of 6-MP intolerance in a study involving 105 Chinese pediatric patients with ALL. The reported minor allele frequency for this variant was 15.7%, with the T allele showing a strong association with reduced dose intensity (DI). Genotype-stratified analysis demonstrated a marked gradient in drug tolerance: patients harboring the homozygous TT genotype exhibited pronounced sensitivity, with a median DI of 60.27%, compared to 83.83% in heterozygous (TC) individuals and 94.24% in wild-type (CC) patients. Moreover, the c.415C>T variant was significantly associated with an increased risk of leukopenia (OR = 3.62; *P* = 0.009), with an even stronger effect observed for early-onset leukopenia within the first 60 days of therapy (OR = 9.63; *P* = 3.75 × 10^−4^). Collectively, these findings underscore the clinical relevance of *NUDT15* genotyping and support its implementation as a critical tool for guiding individualized 6-MP dosing strategies in the pediatric population (Zhou et al., 2018). Furthermore, the latest evidence highlights the “additive effects” of carrying multiple risk alleles; patients classified as TPMT/NUDT15 compound intermediate metabolizers (carrying one no-function allele in each gene) tolerate a significantly lower median 6-MP dosage of 25.7 mg/m^2^ compared to intermediate metabolizers for only a single gene (P < 0.001). Based on these diverse findings, updated guidelines recommend adjusting starting doses based on the specific combinations of these genotypes to mitigate fatal toxicities (Maillard et al., 2026).

Importantly, the clinical utility of pharmacogenetic testing has been demonstrated in real-world settings; for instance, at St. Jude Children’s Research Hospital, prospective thiopurine dosing informed by *TPMT* genotype allowed dose reductions without compromising treatment efficacy, supporting the feasibility and clinical benefit of genotype-guided thiopurine dosing (St. Jude Children’s Research Hospital, n. d.).

Beyond these well-established gene *TPMT* and *NUDT15* gene variants associations with thiopurine toxicity, other polymorphisms in metabolic pathways also appear clinically relevant. For example, functional variants in *ITPA*, including rs1127354 (Pro32Thr) and IVS2+21A>C, have been associated with increased susceptibility to mercaptopurine-related adverse effects (Mei et al., 2015). Notably, in a pharmacogenetic multicentric retrospective cohort study involving 508 pediatric and adolescent patients (age between 1 and 17 years old) newly diagnosed with Philadelphia chromosome–negative ALL and treated according to the AIEOP-BFM ALL 2000 protocol, toxicity episodes were graded using the National Cancer Institute Common Terminology Criteria for Adverse Events (NCI-CTC), version 2.0. Within this framework, the *ITPA* rs1127354 homozygous variant genotype was associated with a significantly increased risk of severe gastrointestinal toxicities including nausea, vomiting, diarrhea and stomatitis (OR = 7.73; 95% CI = 1.04–57.34, p = 0.046) as well as neurological adverse events (OR = 13.23; 95% CI = 1.74–100.65, p = 0.013) during the induction and consolidation phases, based on multivariable analyses adjusting for clinical confounders (Franca et al., 2017). These findings suggest a potential role of *ITPA* variation in modulating thiopurine tolerance beyond hematologic toxicity, although its clinical utility remains less well established and is not currently incorporated into routine pharmacogenetic dosing guidelines (Franca et al., 2017). Moreover, in a retrospective cohort study involving 210 Middle Eastern children with ALL, Moradveisi et al. identified the *ITPA* rs7270101 (c.124+21A>C) variant as a significant predictor of 6-MP intolerance and hematological toxicity. The researchers found that carrying one or two *ITPA* risk alleles was significantly associated with a higher incidence of febrile neutropenia (P < 0.001) across the combined cohorts (Moradveisi et al., 2019).

### 3.1.3 Clinical guideline recommendation

Current international guidelines strongly support genotype-guided dosing of mercaptopurine in ALL. The most significant update comes from the 2024 Clinical Pharmacogenetics Implementation Consortium (CPIC) revision, prompted by the multiethnic pharmacogenomic study by Maillard et al., which demonstrated additive toxicity risk in individuals who are intermediate metabolizers (IM) for both TPMT and NUDT15. Based on these findings, CPIC updated its dosing recommendation: TPMT IM/NUDT15 IM patients should now start at 20%–50% of the standard dose, with titration guided by tolerance and blood counts (Maillard et al., 2024). More recent CPIC updates further emphasize that any genotype combination involving a poor metabolizer (PM) phenotype for either TPMT and/or NUDT15, regardless of the metabolic status of the second gene (normal, intermediate, or indeterminate), confers a markedly elevated risk of severe thiopurine-related toxicity, including leukopenia, neutropenia, and profound myelosuppression. In such high-risk patients, standard dosing may lead to life-threatening or even fatal toxicity if not appropriately adjusted. Consequently, CPIC recommends drastically reduced starting doses, typically on the order of a 10-fold reduction, with reduced frequency to thrice weekly instead of daily, followed by careful titration based on clinical response and hematologic parameters (Table 1) (Clinical Pharmacogenetics Implementation Consortium, 2024; Maillard et al., 2024; Maillard et al., 2026). For single-gene reduced-function phenotypes, CPIC continues to recommend reducing starting dose to 30%–80% of full 6-MP dose for intermediate metabolizers and drastic dose reduction (10% of full dose) or an alternative agent for poor metabolizers, particularly in oncology settings (Clinical Pharmacogenetics Implementation Consortium, 2024; Relling et al., 2019). The Dutch Pharmacogenetics Working Group provides similar guidance: approximately 50% dose reduction for intermediate metabolizers and 10% dosing or substitution for poor metabolizers (Coenen et al., 2026). Clinical oncology frameworks echo these pharmacogenetic recommendations. The National Comprehensive Cancer Network (NCCN)- pediatric ALL guideline advises beginning at 30%–80% of standard dose in TPMT IM patients and substantially lower doses (10%) in PM patients, combined with close hematologic monitoring (National Comprehensive Cancer Network, 2026). Likewise, the FDA label for mercaptopurine recommends major dose reduction in individuals with TPMT or NUDT15 deficiency (Food and Drug Administration (FDA), 2024).

### 3.2 L-asparaginase

L-Asparaginase (L-ASNase) is a bacterial enzyme that catalyzes the hydrolysis of the amino acid *L-asparagine* into *L-aspartic acid* and ammonia. Systemic administration of L-ASNase induces plasma asparagine depletion, thereby impacting malignant lymphoblasts which become unable to synthesize sufficient endogenous asparagine due to low asparagine synthetase activity. This metabolic starvation disrupts protein synthesis and triggers apoptosis in leukemic cells that are highly dependent on extracellular asparagine for growth and survival (Van Trimpont et al., 2022).

Clinical formulations of L-ASNase are primarily derived from *Escherichia coli* or *Erwinia chrysanthemi*. These bacterial-derived preparations form the cornerstone of asparaginase-based therapy and are recognized in the World Health Organization (WHO) Model List of Essential Medicines. Three L-asparaginase formulations are currently used worldwide in the treatment of acute leukemia: native *E. coli* asparaginase, its pegylated form (PEG-asparaginase), and the preparation derived from *Erwinia* chrysanthemi. Native *E. coli* asparaginase was the first to be introduced and, despite its well-known limitations, particularly hypersensitivity reactions and silent inactivation, it remains widely used in low and middle-resource settings because of its lower cost and availability. The development of PEG-asparaginase helped overcome many of these drawbacks; pegylation extends the enzyme’s half-life, reduces immunogenicity, and permits less frequent dosing. For patients who develop allergy or neutralizing antibodies to E. coli–based products, *Erwinia* asparaginase serves as the essential alternative. In developed countries, where native *E. coli* asparaginase has largely been discontinued, the PEG-Asparaginase formulation is now considered the standard substitution (Maese and Rau, 2022).

#### 3.2.1 Mechanism of drug-induced toxicity

The toxicities associated with L-asparaginase can be broadly categorized into immunologic and non-immunologic mechanisms. Immunologic reactions arise from the enzyme’s bacterial origin and large molecular size, which confer high immunogenicity. Consequently, patients may develop hypersensitivity reactions ranging from mild urticaria to severe anaphylaxis. Non-immunologic toxicities are primarily metabolic in nature and stem from the enzyme’s broader catalytic activity on amino acid and nitrogen metabolism. In addition to depleting asparagine, L-asparaginase also hydrolyzes glutamine and, to a lesser extent, aspartate, resulting in the accumulation of neurotoxic by-products such as ammonia, L-aspartic acid, and glutamic acid. Excessive levels of the latter two amino acids can induce excitotoxic neuronal injury in the central nervous system (CNS) by overstimulating *N-methyl-D-aspartate* (NMDA) receptors (Śliwa-Tytko et al., 2022). This triggers massive intracellular calcium influx, mitochondrial dysfunction, and ultimately apoptotic neuronal death. Hyperammonemia further aggravates CNS toxicity by promoting diffuse neuronal depression. Clinically, this may manifest as seizures, altered mental status, or encephalopathy (Fonseca et al., 2021).

Beyond neurotoxicity, L-asparaginase is associated with a spectrum of systemic metabolic adverse effects arising from its interference with protein synthesis and hepatic metabolic pathways. The most prominent manifestation is hepatotoxicity, typically presenting with steatosis and elevations in aminotransferases, and progressing to cholestasis in more severe cases. Perturbations in glucose homeostasis, including treatment-related hyperglycemia and occasional ketoacidosis, further highlight the need for vigilant biochemical monitoring throughout therapy (van der Sluis et al., 2016).

**FIGURE 1:**
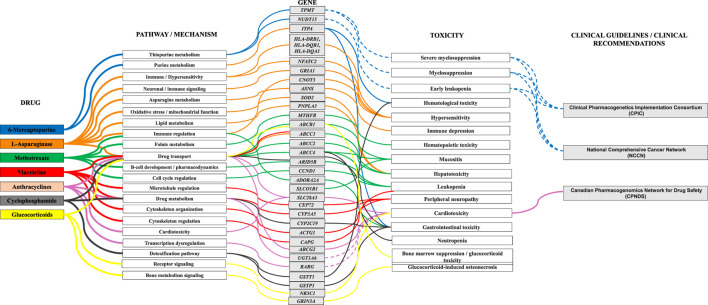
Integrated pharmacogenomic map of leukemia therapeutics: from molecular pathways to clinical toxicities. *TPMT*: Thiopurine S-méthyltransférase; *NUDT15*: Nudix hydrolase 15; *ITPA*: Inosine Triphosphate Pyrophosphatase; *NFATC2*: Nuclear Factor of Activated T-cells, Cytoplasmic, Calcineurin-dependent 2; *GRIA1*: Glutamate Receptor, Ionotropic, AMPA 1; *ASNS*: Asparagine Synthetase; *CNOT3*: CCR4-NOT Transcription Complex Subunit 3; *HLA-DRB1*: Major Histocompatibility Complex, Class II, DR BETA-1; *HLA-DQB1*: Major Histocompatibility Complex, Class II, DQ BETA-1; *HLA-DQA1*: Major Histocompatibility Complex, Class II, DQ ALPHA-1; *SOD2*: Superoxide Dismutase 2; *PNPLA3*: Patatin-Like Phospholipase Domain-Containing Protein 3; *MTHFR*: Methylenetetrahydrofolate reductase; *ABCB1*: ATP-binding cassette subfamily B member 1; *ABCC1*: ATP-binding cassette sub-family C member 1; *ABCC2*: ATP-Binding Cassette, Subfamily C, Member 2; *ABCC4*: ATP-binding cassette sub-family C member 4; *ARID5B*: AT-Rich Interaction Domain 5B; *CCND1*: Cyclin D1; *ADORA2A*:Adenosine A2A Receptor; *SLCO1B1*: Solute Carrier Organic Anion Transporter family member 1B1; *SLC28A3*: Solute Carrier Family 28 Member 3; *CEP72*: Centrosomal Protein, 72-KD; *CYP3A5*: Cytochrome P450, Subfamily IIIA, Polypeptide 5; *CYP2C19*: Cytochrome P450 Family 2 Subfamily C Member 19; *ACTG1*: Actin, Gamma-1; *CAPG*: Capping Protein, Gelsolin-Like; *ABCG2*: ATP-Binding Cassette, Subfamily G, Member 2; *UGT1A6*: UDP-glucuronosyltransferase family 1 member A6; *RARG*: Retinoic Acid Receptor Gamma; *GSTT1*: Glutathione S-Transferase Theta 1; *GSTP1*: Glutathione S-Transferase Pi 1; *NR3C1*: Nuclear Receptor Subfamily 3 Group C Member 1; *GRIN3A*: Glutamate Ionotropic Receptor NMDA Type Subunit 3A.

Recent mechanistic and pharmacogenetic investigations have begun to elucidate the molecular pathways underpinning asparaginase-associated hepatotoxicity. Experimental and clinical observations suggest that the enzyme triggers an amino-acid stress response, characterized by profound intracellular depletion of asparagine and glutamine. This metabolic perturbation disrupts cellular redox homeostasis and promotes excessive generation of reactive oxygen species (ROS), ultimately leading to oxidative injury and hepatocellular apoptosis. Within this context, several molecular determinants have been implicated in modulating susceptibility to hepatic toxicity, including the mitochondrial antioxidant enzyme superoxide dismutase 2 (*SOD2*), the lipid-metabolism-related protein patatin-like phospholipase domain-containing 3 (*PNPLA3*) and the ATP-binding cassette transporter *ABCC1*, which participates in the efflux of xenobiotics and oxidative metabolites. Functionally, the superoxide dismutase enzymatic system constitutes a key cellular defense against oxidative stress by catalyzing the conversion of superoxide radicals into molecular oxygen and hydrogen peroxide, thereby limiting ROS-mediated damage. Genetic variability affecting these pathways may therefore influence individual vulnerability to asparaginase-induced hepatotoxicity and has emerged as an area of increasing interest in pharmacogenomic studies of pediatric ALL (Figure 1) (Aslam et al., 2021).

#### 3.2.2 Genetic variants associated with L-asparaginase toxicity

Genetic predisposition is increasingly recognized as a major determinant of L-asparaginase–induced hypersensitivity in ALL (ALL). Multiple genome-wide, exome-wide, and candidate-gene studies have explored both HLA and non-HLA variants associated with asparaginase toxicity (Lopez-Santillan et al., 2017), with all reported associations currently classified as Level 3 evidence in ClinPGX. Among HLA genes, HLA-DRB1*07:01, with an allele frequency below 13% globally, has been the most consistently linked to hypersensitivity risk, underscoring the pivotal role of antigen presentation pathways in mediating immune recognition of bacterial asparaginase, with additional associations reported for HLA-DQB1*02:02 and HLA-DQA1*02:01 (Gagné et al., 2020). Furthermore, the HLA-DRB1*07:01-DQB1*02:02 haplotype carriers were positively and significantly associated with an increased risk to asparaginase hypersensitivity (Table 2) (Pavlovic et al., 2019).

**TABLE 2:** Genetic variants associated with L-Asparaginase toxicity.

Toxicity phenotype	Gene	Variant (rsID/HGVS nomenclature)	Study design/Genetic approach	Sample size, OR/HR (95% CI); p value)	References	Allele frequency in reference populations
Hypersensitivity	*HLA-DRB1*	HLA-DRB1*07:01	Retrospective cohort study/genetic association study	N = 284, OR = 2.2 (95% IC: 1.2–4.3); p = 0.01	Gagné et al. (2020)	Global: 13% European: 13% African: 13% East Asian: 0.00%
	*HLA-DQB1*	HLA-DQB1*02:02	Retrospective cohort study/genetic association study	N = 284, OR = 2.6 (95% IC: 1.3–5.2); p = 0.006		European: 10% North African: 17% Southeast Asian: 4%
	*HLA-DQA1*	HLA-DQA1*02:01	Retrospective cohort study/genetic association study	N = 284, OR = 4.9 (95% IC: 1.1–22.8); p = 0.04		European: 15% North African: 18% Southeast Asian: %
Hypersensitivity	*NFATC2*	NM_012340.5: c.2722+12,367 T>A (rs6021191)	Prospective cohort study/genome-wide association study (GWAS)	N = 3,308, OR = 3.11 (95% IC: 2.07–4.66); p = 4.1 × 10^−8^	Fernandez et al. (2015)	Global: 4.86% European: 0.11% African: 14.13% East Asian: 12.16%
Hypersensitivity	*GRIA1*	NM_001364165.2: c.2271-3807G>A (rs4958351)	Prospective cohort study/genome-wide association study (GWAS)	N = 485, HR = 1.75 (95% CI: 1.41–2.17); p = 3.5 × 10^−7^	Chen et al. (2010)	Global: 6.48% European: 4.13% African: 9.95% East Asian: 11.56%
			Retrospective cohort/candidate gene association study	N = 146, OR = 1.74 (95% IC: 0.389–0.845); p = 0.003	Rajić et al. (2015)	
		NM_001364165.2: c.2270+2302G>A (rs4958676)	Prospective cohort study/genome-wide association study (GWAS)	N = 485, HR = 1.56 (95% CI: 1.25–1.95); p = 9.2 × 10^−5^	Chen et al. (2010)	Global: 17.64% European: 19.36% African: 16.64% East Asian: 0.42%
			Retrospective cohort/candidate gene association study	N = 146, OR = 1.59 (95% IC: 0.460–0.857); p = 0.005	Rajić et al. (2015)	
		NM_001364165.2: c.1824–20561C>G (rs6889909)	Prospective cohort study/genome-wide association study (GWAS)	N = 485, R = 1.51 (95% CI: 1.21–1.87); p = 2.3 × 10^−4^	Chen et al. (2010)	Global: 18.09% European: 19.56% African: 18.27% East Asian: 0.17%
			Retrospective cohort/candidate gene association study	N = 146,OR = 1.59 (95% IC: 0.460–0.857); p = 0.005	Rajić et al. (2015)	
		NM_001364165.2: c.2386–3102C>T (rs10070447)	Prospective cohort study/genome-wide association study (GWAS)	N = 485, HR = 1.66 (95% CI: 1.35–2.05); p = 2.0 × 10^−6^	Chen et al. (2010)	Global: 30.42% European: 35.05% African: 25.81% East Asian: 3.19%
			Retrospective cohort/candidate gene association study	N = 146, OR = 1.65 (95% IC: 0.408–0.899); p = 0.006	Rajić et al. (2015)	
		NM_001364165.2: c.1824–20506C>T (rs6890057)	Prospective cohort study/genome-wide association study (GWAS)	N = 485, HR = 1.63 (95% CI: 1.30–2.05); p = 2.1 × 10^−5^	Chen et al. (2010)	Global: 17.79% European: 19.35% African: 17.77% East Asian: 0.17%
			Retrospective cohort/candidate gene association study	N = 146, OR = 1.59 (95% IC: 0.460–0.857); p = 0.005	Rajić et al. (2015)	
Immune depression	*ASNS*	NM_183356.4: c.-60+27_-60+40dup (rs3832526)	Retrospective cohort study/candidate gene association study	N = 285, OR = 14.6 (95% IC: 3.6–58.7); p < 0,0005	Ben Tanfous et al. (2015)	Global: 23% European: 12% African: 21% East Asian: 29%
			Observational prospective study/candidate gene association study	N = 88, OR = 0.50 (95% CI: 0.12–2.08); p = 0.340	Youssef et al. (2021)	
Hypersensitivity	*CNOT3*	NM_014516.4: c.1606–983T>C (rs73062673)	Prospective cohort study/GWAS	N = 831, OR = 3.73 (95% CI: 2.33–5.98); p = 4.68 × 10^−8^	Højfeldt et al. (2019)	Global: 12% European: 12% African: 4% East Asian: 6%
Hepatotoxicity	*SOD2*	NM_000636.4: c.47 T>C (rs4880)	Retrospective cohort study/candidate gene association study	N = 300, OR = 7.82 (95% IC: 3.86–15.85); p < 0.05	Aslam et al. (2021)	Global: 47.05% European: 50.33% African: 42.46% East Asian: 14.36%
Hepatotoxicity	*PNPLA3*	NM_025225.4: c.444C>G (rs738409)	Retrospective cohort study/candidate gene association study	N = 300. OR = 5.82 (95% CI: 3.43–9.87); p < 0.05	Aslam et al. (2021)	Global: 22.83% European: 22.62% African: 13.97% East Asian: 38.67%
Hepatotoxicity	*ABCC1*	NM_004996.4: c.132044G>T (rs4148350)	Retrospective cohort study/candidate gene association study	N = 300, OR = 2.52 (95% IC: 1.55–4.10); p < 0.05	Aslam et al. (2021)	Global: 7.43% European: 5.84% African: 10.83% East Asian: 5.00%

*HLA-DRB1*, major histocompatibility complex, Class II, DR BETA-1; *HLA-DQB1*, major histocompatibility complex, Class II, DQBETA-1; *HLA-DQA1*, major histocompatibility complex, Class II, DQ ALPHA-1; *SOD2*, Superoxide Dismutase 2; *PNPLA3*, Patatin-Like Phospholipase Domain-Containing Protein 3; *ABCC1*, ATP-binding cassette sub-family C member 1; N/A, Not Available. No clinical guidelines or recommendations are currently applicable to any of the reported variants. Allele frequencies in reference populations were obtained from the Genome Aggregation Database (gnomAD), HLA allele frequencies were obtained from the allele frequency Net Database (https://doi.org/10.1093/nar/gkz1029).

Beyond the HLA locus, several non-HLA genes have been implicated in modulating hypersensitivity risk, including *GRIA1*, *NFATC2*, and *ASNS*. These associations, while biologically plausible, remain heterogeneous and inconsistently replicated—likely reflecting modest effect sizes, limited cohort sizes, differences in phenotype definition, and ancestry-specific allele frequencies (Anderson, 2025). Among this non-HLA loci, the *NFATC2* variant rs6021191 has shown one of the strongest associations with hypersensitivity (Fernandez et al., 2015). This allele displays marked population stratification, occurring approximately three times more frequently in individuals of African ancestry compared with the general population (14.13% vs. 4.86%) and being virtually absent in Europeans, highlighting population-specific genetic risk (Anderson, 2025). Another repeatedly replicated marker is the *GRIA1* variant rs4958351, initially identified as a susceptibility factor for hypersensitivity, alongside additional *GRIA1* variants, rs4958676, rs6889909, rs6890057, and rs10070447, which remain under investigation for their contribution to immune-mediated adverse reactions (Rajić et al., 2015; Chen et al., 2010). Several of these alleles also exhibit higher frequencies in African populations, reaching approximately 10%. Variants in the *ASNS* gene, which encodes asparagine synthetase may contribute to interindividual differences in toxicity. In particular, the *ASNS* polymorphism rs3832526 and the 3-repeat (3R) homozygous genotype have been associated with increased hypersensitivity, likely through altered intracellular asparagine availability influencing immune activation (Ben Tanfous et al., 2015; Youssef et al., 2021).

In contrast to native *E. coli* asparaginase, PEG-asparaginase (Oncaspar) displays a distinct immunogenetic profile. Pegylation masks bacterial epitopes, reducing direct antibody recognition and thereby altering the genetic determinants of hypersensitivity. A genome-wide association study identified the *CNOT3* intronic variant rs73062673 as significantly associated with PEG-asparaginase allergy (Højfeldt et al., 2019). The risk of hypersensitivity increases in a dose-dependent manner with the number of risk alleles across implicated loci and a significant interaction has been demonstrated between *CNOT3* and HLA-DQA1*. Individuals carrying multiple *CNOT3* and HLA-DQA1* risk alleles exhibit a substantially higher cumulative incidence of PEG-asparaginase hypersensitivity (Højfeldt et al., 2019).

In addition to hypersensitivity-related loci, pharmacogenetic studies have also identified variants associated with hepatotoxicity in pediatric patients treated with asparaginase-containing chemotherapy. Notably, the minor G allele of *SOD2* rs4880 has been associated with an increased risk of hepatic toxicity (OR = 2.63; 95% CI: 1.42–4.84), with a stronger effect observed under a recessive genetic model (OR = 7.82; 95% CI: 3.86–15.85) (Aslam et al., 2021). This variant in the superoxide dismutase 2 (*SOD2*) gene has also been associated with a higher incidence of asparaginase-related hepatotoxicity in adult cohorts, primarily among White non-Hispanic patients with ALL (Wu Y. et al., 2022). Similarly, the *ABCC1* rs4148350 polymorphism has been significantly associated with treatment-related hepatotoxicity under a dominant genetic model (OR = 2.52; 95% CI: 1.55–4.10), with both heterozygous and homozygous variant genotypes contributing to the increased risk (Aslam et al., 2021). The prevalence and genetic susceptibility to hepatotoxicity among ALL patients undergoing induction chemotherapy motivated liver function tests evaluation to better monitor and manage patients in the future (Mekonnen and Wondmeneh, 2022).

#### 3.2.3 Clinical guidelines

Currently, no clinical guidelines recommend routine pre-treatment genetic testing to predict hypersensitivity or toxicity to L-asparaginase. There is still no recommendation for upfront testing to anticipate toxicity before treatment initiation, mainly the low level of evidence of here enumerated associations. Management of asparaginase therapy relies on therapeutic drug monitoring (TDM) (Baruchel et al., 2020), and measurement of serum asparaginase activity remains the most reliable surrogate marker of therapeutic efficacy.

### 3.3 Methotrexate

Methotrexate (MTX) is an antifolate drug, it functions in leukemia cells essentially as a competitive inhibitor of dihydrofolate reductase (DHFR), preventing the conversion of dihydrofolate to tetrahydrofolate, thereby depleting reduced folate, an essential cofactor for nucleotide synthesis which results in locking thymidine and novo purine synthesis (DNPS) key elements in DNA duplication and cell survival resulting in cell death (Cronstein, 2015).

Cellular uptake of MTX occurs *via* human reduced folate carrier 1(*SLC19A1*). Inside the cell MTX undergoes a critical activation by polyglutamination catalyzed by folylpolyglutamate synthetase (FPGS) which enhances intracellular retention and the inhibitory effect maximizing the therapeutic effect (Kato et al., 2012).

As a result of the impaired cellular development in healthy cells as well as in tumor cells, MTX has some major and serious side effects such as mucositis and cytopenia leading to increased bleeding, easily bruising, and an increased risk of infections.

#### 3.3.1 Mechanism of drug-induced toxicity

Methotrexate (MTX)–induced hematological toxicity is multifactorial, and several hypotheses have been proposed. One widely accepted mechanism is the inhibition of DNA synthesis, leading to impaired DNA replication in rapidly dividing cells such as bone marrow progenitor cells. Additionally, some studies have shown that MTX-induced thrombocytopenia is mediated by increased platelet apoptosis, which is associated with mitochondrial dysfunction (Paul et al., 2015). Furthermore, cells with a high capacity for polyglutamination, such as leukemic myeloblasts, lymphoblasts, and epithelial cells, are more susceptible to MTX toxicity. Polyglutamination prolongs intracellular drug retention, which enhances cytotoxic effects and contributes to the development of adverse side effects (Colebatch et al., 2011).

#### 3.3.2 Genetic variants associated with methotrexate toxicity

Methylenetetrahydrofolate reductase (*MTHFR*) is one of the most extensively studied genes involved in methotrexate (MTX) metabolism. The single-nucleotide polymorphisms (SNPs) 677C>T (rs1801133) and 1298A>C (rs1801131) are both associated with reduced enzyme activity and have been significantly linked to an increased risk of hematopoietic toxicity in patients treated with MTX (Han et al., 2021). This elevated risk may be attributable, at least in part, to increased circulating MTX concentrations. Moreover, in Lebanese children with ALL, carriers of the *MTHFR* rs1801133 variant allele were found to have a statistically significant threefold increased risk (OR = 3.057) of experiencing a decrease in hemoglobin levels during the consolidation phase of methotrexate therapy (Zgheib et al., 2014). Gemmati *et al.* analyzed 110 patients with non-Hodgkin’s lymphoma treated with MTX and reported that, for the *MTHFR* 677C>T polymorphism, the TT genotype was associated with an increased risk of mucositis (Gemmati et al., 2007). The implication of these polymorphisms in MTX toxicity has been supported by the result of a metaanalysis conducted in 2012 (Yang et al., 2012). Nevertheless, a recent metaanalysis concluded that there is no association between the *MTHFR*variant and toxicity (Lopez-Lopez et al., 2013).

SLCO1B1, an organic anion transporter, mediates MTX uptake into hepatocytes and renal cells for metabolism and excretion. Minor alleles of SNPs in the *SLCO1B1* gene can alter transporter activity, leading to changes in MTX clearance. For example, for the SNP rs4149081 (A>G), patients with the GG genotype have a higher risk of mucositis when treated with MTX (Treviño et al., 2009). A similar association has been reported for carriers of the C allele of rs11045879 (T>C), patients with C allele have decreased clearance of MTX which is linked to a higher chance of gastrointestinal toxicity (Yang et al., 2022). Other transporter gene polymorphisms have also been associated with MTX toxicity. For the *ABCB1* SNPs 1236C>T and 3435C>T, patients carrying the TT genotype exhibit a higher incidence of mucositis (Gong et al., 2021).

In contrast to these results, the polymorphism rs7317112 (A>G) of the *ABCC4* gene has a protective effect. In fact the genotype GG is associated with a decreased risk for mucositis when treated with MTX (den Hoed et al., 2015). This association may be explained by the enhanced MTX clearance observed in GG genotype carriers among pediatric ALL patients (Braidotti et al., 2025).

The *ABCC2* gene has been implicated in an increased risk of leukopenia and hepatotoxicity. Specifically, for the SNP 154A>G (rs3740065), patients with the AG or GG Genotype show a higher risk of hepatotoxicity compared with those carrying the AA genotype (Zhao et al., 2021; Gong et al., 2021). In addition, for rs717620, carriers of the T allele have an increased risk of leukopenia (Razali et al., 2020; Gong et al., 2021).

For the *CCND1* gene, the SNP rs9344 known as G870A is highly associated with a lower incidence of leukopenia, thrombocytopenia and liver toxicity compared to the AA genotype when treated with MTX (Zhao et al., 2021; Gong et al., 2021). However, this association was only found in one study conducted on a small cohort suggesting that further investigation is needed.

*ADORA2A* rs2236624 homozygous mutated genotypes were associated to neurotoxicity and hepatotoxicity, with induction-consolidation ALL-treatment including Methotrexate, in retrospective study in children (Franca et al., 2017). Interestingly, the same variant gene (rs2236624) was associated with increased methotrexate gastrointestinal events likely by altering adenosine receptor signaling and enhancing susceptibility to gastrointestinal adverse effects. In the same study, *ABCC1* rs246240 variant was associated with increased methotrexate toxicity, likely by reducing drug efflux capacity and leading to higher intracellular methotrexate exposure (Figure 1; Table 3) (Franca et al., 2017).

**TABLE 3:** Genetic variants associated with methotrexate toxicity.

Toxicity phenotype	Gene	Variant (rsID/HGVS nomenclature)	Study design/Genetic approach	Sample size, OR/HR, (95% CI); p value	References	Allele frequency in reference populations
Hematopoietic toxicity	*MTHFR*	NM_005957.5: c.665C>T (rs1801133)	Cohort study, candidate gene association study	N = 127, OR = 3.057 (95% CI: 1.217; 7.680)	Zgheib et al. (2014)	Global: 27.54% European: 33.73% Africain: 11.21% East Asian: 30.22%
			Retrospective cohort study/candidate gene association study	N = 157, p = 0.003	Han et al. (2021)	
		NM_005957.5: c.1286A>C (rs1801131)	Retrospective cohort study/candidate gene association study	N = 129, OR = 2.78 (95% CI: 1.26–6.13); p = 0.011	Salazar et al. (2024)	Global: 26.34% European: 31.59% Africain: 16.7% East Asian: 22.05%
Mucositis	*SLCO1B1*	NM_006446.5: c.1865+248G>A (rs4149081)	Genome-wide association study (GWAS), candidate gene association study	N = 640, (95% CI: 7.2–13.7); p = 6.7 × 10^−10^	Treviño et al. (2009)	Global: 18.57% European: 17.11% Africain: 15.84% East Asian: 45.12%
		NM_006446.5: c.1865+4846T>C (rs11045879)	Genome-wide association study (GWAS), candidate gene association study	N = 640, (95% CI: 6.7–14); p = 8.2 × 10^−11^	Treviño et al. (2009)	Global: 18.56% European: 17.12%
Mucositis	*ABCB1*	NM_001348946.2: c.1236T>C (rs1128503)	Prospective cohort study/candidate gene association study	N = 103, p < 0.05	Gurieva et al. (2023)	African: 15.85% East Asian: 45.02%
			Retrospective cohort study/candidate gene association study	N = 80, OR = 3.04 (95% CI: 1.39–6.64); p = 0,0022	Gong et al. (2021)	​
		NM_001348946.2: c.2677C>T (rs1045642)	Retrospective cohort study, candidate gene association study	N = 80, OR = 2.38 (95% C I: 1.15–5.00); p = 0,0052	Gong et al. (2021)	Global: 42.81% European: 53.12% African: 20.30% East Asian: 38.04%
Hepatotoxicity	*ABCC1*	NM_004996.4: c.616–7942A>G/T (rs246240)	Retrospective cohort study/candidate gene association study	N = 508, OR = 2.25 (95% CI: 1.03–4.88); p = 0.041	Franca et al. (2017)	Global: 20.05% European: 24.88% African: 5.14% East Asian: 35.21%
Hepatotoxicity	*ABCC2*	NM_000392.5: c.4146+154A>G (rs3740065)	Retrospective cohort study/candidate gene association study	N = 65, OR = 3.14 (95% CI: 1.89–5.20); p = 0.00001	Zhao et al. (2021)	Global: 15.14% European: 10.57% African: 21.47% East Asian: 33.19%
			Retrospective cohort study/candidate gene association study	N = 80, OR = 0.71 (95% CI: 0.34–1.47); p = 0.34	Gong et al. (2021)	
Leucopenia		NM_000392.5: c.-24C>T (rs717620)	Prospective cohort study/candidate gene association study	N = 38, p = 0.03	Razali et al. (2020)	Global: 15.49% European: 19.85% African: 6.16% East Asian: 20.69%
			Retrospective cohort study, candidate gene association study	N = 80, OR = 1.73 (95% CI: 0.70–4.27); p = 0.24	Gong et al. (2021)	
Mucositis	*ABCC4*	NM_005845.5: c.75–23516T>G (rs7317112)	Prospective cohort study/candidate gene association study	N = 134, p = 0.016	den Hoed et al. (2015)	Global: 36.51% European: 27.82% African: 59.34% East Asian: 33.84%
			Retrospective cohort study/candidate gene association study	N = 175, p = 0,0000206	Braidotti et al. (2025)	
Leucopenia	*ARID5B*	NM_032199.3: c.734–5030T>C (rs4948496)	Prospective cohort study/candidate gene association study	N = 38, p = 0.02	Razali et al. (2020)	Global: 55.27% European: 45.99% African: 65.69% East Asian: 64.21%
Leucopenia	*CCND1*	NM_053056.3: c.723G>A (rs9344)	Retrospective cohort study/candidate gene association study	N = 65, OR = 0.42 (95% CI: 0.24–0.73); p = 0.0023	Zhao et al. (2021)	Global: 39.26% European: 45.85% African: 21.88% East Asian: 54.58%
			Retrospective cohort study/candidate gene association study	N = 80, OR = 0.75 (95% CI: 0.37–1.50); p = 0.41	Gong et al. (2021)	
Gastrointestinal	*ADORA2A*	NM_001278497.2: c.333–527T>C (rs2236624)	Retrospective cohort study/candidate gene association study	N = 508, OR = 2.25 (95% CI: 1.03–4.88); p = 0.041	Franca et al. (2017)	Global: 15.34% European: 15.47% African: 8.58% East Asian: 37.31%

*MTHFR*, Methylenetetrahydrofolate reductase*, SLCO1B1*, Solute Carrier Organic Anion Transporter family member 1B1*; ABCB1*, ATP-binding cassette sub-family B member 1; *ABCC1*, ATP-binding cassette sub-family C member 1; *ABCC2*, ATP-Binding Cassette, Subfamily C, MEMBER, 2; *ABCC4*, ATP-binding cassette sub-family C member *4; ARID5B*, *AT-Rich Interaction Domain 5B; CCND1*, *Cyclin D1; ADORA2A:*Adenosine A2A Receptor. N/A, Not Available. No clinical guidelines or recommendations are currently applicable to any of the reported variants. Allele frequencies in reference populations were obtained from the Genome Aggregation Database (gnomAD).

### 3.4 Anthracycline

Anthracyclines are a class of chemotherapeutic agents originally derived from *Streptomyces* species. Among them, daunorubicin and doxorubicin exert their antileukemic effects by intercalating into DNA and inhibiting the enzyme topoisomerase II, thereby disrupting DNA replication, and ultimately inducing cell death (Moiseeva, 2019; Venkatesh and Kasi, 2025). In the treatment of ALL, anthracyclines are commonly administered during the induction phase and the early consolidation/intensification phases of therapy. In combination with other chemotherapeutic agents, they contribute significantly to achieving high remission rates (Venkatesh and Kasi, 2025).

Despite their critical role in ALL therapy, anthracycline-induced cardiotoxicity (ACT) remains a major long-term adverse drug reaction that limits their clinical use and is associated with substantial morbidity and mortality.

#### 3.4.1 Mechanism of drug-induced toxicity

Anthracyclines cardiotoxicity (ACT) is the result of several mechanisms: Topoisomerase IIβ inhibition occurs not only in leukemia cells but also in mature cardiomyocytes inducing DNA damage in cardiac tissue, leading to cardiomyocyte apoptosis (Zhang et al., 2012).

Oxidative stress is also an important mechanism in anthracycline-induced cardiotoxicity, in fact Doxorubicin undergoes reduction-oxidation reactions generating excessive ROS that overwhelm cardiac antioxidant defense system and cause lipid peroxidation, protein oxidation and genomic damage. Additionally, doxorubicin accumulates in mitochondria and binds to cardiolipin, blocks electron transport chain, reducing ATP production, impairing respiration, and amplifying ROS. This sustained oxidative damage eventually leads to mitochondrial dysfunction and irreversible myocardial injury (McGowan et al., 2017).

Doxorubicin also disrupts calcium homeostasis, it impairs calcium regulation through Suppressing NCX and SERCA2a, leading to accumulation of excessive cytosolic calcium. This excess activates calpain proteases that degrades contractile proteins like titin and troponin I, compromising contractility and inducing apoptosis. At the same time mitochondrial calcium uptake accelerates energy depletion and cardiomyocyte death (Qiu et al., 2023).

#### 3.4.2 Genetic variants associated with anthracycline toxicity

Genetic factors play an important role in susceptibility to ACT development, and two genes have been especially implicated in pediatric populations: *SLC28A3* and *RARG* (Figure 1). SLC28A3 is an uptake transporter capable of importing anthracyclines into cardiac cells. *In vitro*, the *SLC28A3* rs7853758 (G>A) variant has been shown to protect cells against doxorubicin-induced cardiotoxicity (Magdy et al., 2022). A highly significant association of the AA allele of *SLC28A3* rs7853758 with decrease of ACT was identified in a cohort of anthracycline-treated children including ALL patients (N = 218, OR = 0.46 (95% CI: 0.20–1.80); p = 0.058) (Visscher et al., 2013). This protective polymorphism was also reported in a Mexican pediatric children cohort as highly associated with decreased ACT (Vargas-Neri et al., 2022). A Canadian study reported that this protective effect of the SLC28A3 rs7853758 variant was significant only at higher doses (>250 mg/m^2^) (Siemens et al., 2023a). It should be noted that both studies focused on heterogeneous cohorts of pediatric cancers, including ALL. Moreover, SLC28A3 rs7853758 variant is consistently identified as protective against cardiomyopathy in childhood cancer survivors, with studies reporting a significantly reduced risk of cardiotoxicity characterized by an OR of 0.36 (95% CI: 0.22–0.60; p = 1.6 × 10^−5^) in a candidate gene analysis and an OR of 0.31 (95% CI: 0.16–0.60; p = 1.0 × 10^−4^) in a genome-wide association study (Aba et al., 2025). Same applies to *SLC28A3* rs885004 (G>A), the AA genotype is highly associated with decreased risk ACT (Aslam et al., 2021). In contrast, the drug transporter pump (*ABCG2*) of the ATP-binding cassette (ABC) family are involved in causing anthracycline related cardiotoxicity (Sharif et al., 2025). Heterozygous *ABCG2* variant rs2231142 was associated with cardiotoxicity in pediatric retrospective study following UKALL 2003 protocol induction treatment [OR 5.25 (1.84–14.95), P = < 0.05] (Aslam et al., 2021).

With a lower impact, *UGT1A6* rs17863,783 has been associated with ACT in the pediatric population (Visscher et al., 2013). *UGT1A6* is not directly involved in anthracyclines detoxification but some of Daunorubicin and doxorubicin and metabolites undergo glucuronidation (Andrews et al., 1980). Impaired *UGT1A6*-mediated glucuronidation of anthracycline metabolites, especially in carriers of the UGT1A6*4 allele may lead to the accumulation of toxic metabolites, increasing the risk of ACT. Retinoic Acid Receptor Gamma (*RARG*) is a nuclear receptor that regulates transcription of genes involved in cardiomyocyte differentiation, survival, and metabolism (Stefanovic and Zaffran, 2017). The *RARG* variant rs2229774 implication in ACT has been discovered in a Genome-wide association study (Aminkeng et al., 2015) and later strongly associated with an increased risk of ACT in children treated with anthracyclines (Siemens et al., 2023b). A proposed mechanism is that the polymorphism alters RARG’s ability to regulate its target genes, which include genes involved in DNA repair and oxidative stress management, as a result, cardiomyocytes become more sensitive to toxicity (Magdy et al., 2022). Another interesting polymorphism association discovered by large-scale association study performed by Wang et al. is concerning *HAS3* gene: the *HAS3* rs2232228 AA genotype patients had increased risk for cardiomyopathy when exposed to high-dose (>250 mg/m2) anthracyclines. Indeed, *HAS3* gene encodes hyaluronan, a component of the extracellular matrix that exhibits antioxidant activity and may play a role in cellular recovery following ROS-mediated cardiac injury (Wang et al., 2013).

Concerning calcium homeostasis pathway, no genetic variants of *NCX* and *SERCA2a* had evidence for drug-induced toxicity, most studies focus on cardiac function, arrhythmia phenotypes or heart failure contexts (Kim et al., 2012; Sikkel et al., 2014), but their role in cardiomyocyte calcium regulation suggests that further investigation could be valuable.

### 3.4.3 Clinical guidelines

No CPIC recommendations for anthracyclines have been published. However, the Canadian Pharmacogenomics Network for Drug Safety (CPNDS) clinical recommendation group has published genotype-based guidelines for anthracyclines (Level B: moderate recommendation), including increasing frequency of echocardiographic monitoring and prescribing derazoxane; CPNDS recommends pharmacogenomic testing of *RARG* rs2229774, *SLC28A3* rs7853758 and UGT1A6*4 (Table 4) (Aminkeng et al., 2016).

**TABLE 4:** Genetic variants associated with Anthracyclines toxicity.

Toxicity phenotype	Gene	Variant (rsID/HGVS nomenclature)	Study design/Genetic approach	Sample size, OR/HR, (95% CI); p value	References	Allele frequency in reference populations	Clinical guideline/Clinical recommendations
Cardiotoxicity	*SLC28A3*	NM_001199633.2: c.1381C>T (rs7853758)^a^	Cohort study, candidate gene association study	N = 454, OR = 0.12 (95% CI: 0.02–0.72); p = 0.009	Aminkeng et al. (2016)	Global: 18.69% European: 13.98% African: 30.78% East Asian: 14.88%	Canadian pharmacogenomics network for drug safety (CPNDS)
				N = 218, OR = 0.46 (95% CI: 0.20–1.80); p = 0.058	Visscher et al. (2013)		
				N = 800, OR = 0.25 (95%CI: 0.12–0.52); p = 2 x10^−4^	Loucks et al. (2022)		
				N = 595, OR = 0.43 (95% CI: 0.22–0.78); p = 0,0093	Siemens et al. (2023a)		
				N = 344, OR = 0.36 (95% IC: 0.22–0.60); p = 1.6 × 10^−5^	Aba et al. (2025)		
		NM_001199633.2: c.862–360C>T (rs885004)^a^	Cohort study, candidate gene association study	N = 218, OR = 0.42 (95% CI: 0.16–1.10); p = 0.058	Visscher et al., 2013,	Global: 11.91% European: 12.47% African: 10.22% East Asian: 14.91%	N/A
	*UGT1A6*	UGT1A6*4 NM_021027.3 c.627G>A (rs17863,783)	Cohort study, candidate gene association study	N = 344, OR = 6.2 (95% CI: 2.5–15.4); p = 1.1 × 10^−4^	Aba et al. (2025)	Global: 5.42% European: 2.03% African: 11.58% East Asian: 2.87%	Canadian Pharmacogenomics Network for drug safety (CPNDS)
	*RARG*	NM_000966.6: c.1280C>T (rs2229774)	Genome-wide association study (GWAS), cohort study/candidate gene association study	N = 344, OR = 4.7 (95% IC: 2.7–8.3); p = 5,9 × 10^−8^	Aba et al. (2025)	Global: 7.31% European: 7.03% African: 8.51% East Asian: 0.37%	Canadian Pharmacogenomics network for drug safety (CPNDS)
	*ABCG2*	NM_004827.3: c.421C>T (rs2231142)	Retrospective cohort study, candidate gene association study	N = 376, OR = 8.45 (95% CI: 4.91–14.52); p < 0.0001	Sharif et al. (2025)	Global: 0.1119% European: 0.1109% African: 0.02677% East Asian: 0.2982%	N/A

*SLC28A3,* Solute Carrier Family 28 Member 3*, RARG,* retinoic acid receptor gamma*; UGT1A6,* UDP-glucuronosyltransferase family 1 member A6; *ABCG2,* ATP-Binding Cassette, Subfamily G, Member 2; N/A*,* not available;

a Variant is associated with decrease of toxicity. Allele frequencies in reference populations were obtained from the Genome Aggregation Database (gnomAD).

### 3.5 Vincristine

As a microtubule-targeting agent, Vincristine is an essential component of frontline therapy for ALL and is delivered across multiple treatment phases, resulting in substantial cumulative exposure. Its narrow therapeutic index and high propensity to induce peripheral neuropathy (VIPN) frequently necessitate dose reductions, thereby highlighting the clinical relevance of inter-individual susceptibility, including ethnic and genetic determinants (Renbarger et al., 2008; Velasco et al., 2010).

#### 3.5.1 Mechanism of drug-induced toxicity and hypersensitivity

Vincristine binds tubulin, disrupting microtubule dynamics, impairing mitotic spindles, and causing cell cycle arrest (Jordan and Wilson, 2004; Kavallaris, 2010). This disruption also impairs axonal transport, the movement of mitochondria and vesicles along microtubules, leading to mitochondrial dysfunction and accumulation of reactive oxygen species (ROS), ultimately triggering SARM1-dependent axon degeneration (Gomez-Deza et al., 2023; LaPointe et al., 2013) and resulting in vincristine-induced peripheral neuropathy (VIPN). Inflammatory pathways also contribute to VIPN. Activation of the NLRP3 inflammasome in macrophages promotes IL-1β release, and inhibition of this pathway with MCC950 or the IL-1 receptor antagonist anakinra prevents neuropathy without affecting antitumor efficacy (Starobova et al., 2021).

Host susceptibility is further influenced by genetic and pharmacokinetic factors. Variants in drug-metabolizing enzymes and transporters, including CYP3A isoforms and ABC transporters, may modulate vincristine exposure and toxicity risk. Additionally, reduced CEP72 expression increases neuronal sensitivity to microtubule destabilization (Figure 1) (Diouf et al., 2015; Klumpers et al., 2022).

#### 3.5.2 Genetic variants associated with vincristine-induced neurotoxicity

Genetic polymorphisms in *CYP3A5* have been consistently associated with interindividual variability in vincristine-induced peripheral neuropathy (VIPN). In an Egyptian cohort of children with rhabdomyosarcoma, genotyping of *CYP3A5* SNPs rs776746, rs10264272, and rs41303343 demonstrated that patients homozygous for the CYP3A5*3/3 genotype had the highest incidence of neuropathy (61.3%), whereas those with CYP3A5*1/*3, CYP3A5*3/*6, and CYP3A5*1/*1 genotypes exhibited lower frequencies (22%, 10.7%, and 4.7%, respectively); the CYP3A5*1/*6 genotype was associated with the lowest risk (Shalaby et al., 2024; Uittenboogaard et al., 2022). Similar *CYP3A5*-mediated variability in vincristine toxicity has been reported in acute leukemia therapy, supporting the pharmacogenomic relevance of *CYP3A5* across pediatric malignancies. In precursor B-cell ALL, *CYP3A5* expressers (carriers of at least one functional *1 allele) experienced significantly less VIPN than non-expressers (3/3 homozygotes, who lack functional *CYP3A5* activity*)* experienced significantly less VIPN than non-expressers (16% vs. 27% of treatment months, p = 0.0007) (Egbelakin et al., 2011). Expressers also exhibited higher plasma concentrations of the primary vincristine metabolite M1 (p = 0.0004) and lower metabolic ratios ([vincristine]/[M1], p = 0.036); importantly, M1 levels were inversely correlated with neuropathy severity, suggesting that enhanced vincristine metabolism mitigates VIPN risk (McClain et al., 2018). Consistent with these findings, African-American children, among whom *CYP3A5* expression is more prevalent, displayed lower neurotoxicity compared with Caucasian patients. Collectively, these data indicate that *CYP3A5* polymorphisms substantially influence vincristine-related neurotoxicity, with poor metabolizers (e.g., *3/*3) at increased risk. In contrast, the contribution of *CYP3A4* variants remains less well defined, highlighting *CYP3A5* as a promising biomarker for vincristine dosing optimization.

Beyond drug metabolism, variants affecting microtubule dynamics have also been implicated in VIPN susceptibility. The intronic *CEP72* variant rs924607 has been associated with reduced gene expression and increased risk of VIPN in both pediatric and adult ALL patients (Diouf et al., 2015; Stock et al., 2017). Homozygosity for the TT risk genotype has been linked to higher rates of neuropathy, occasionally necessitating vincristine dose reduction or treatment interruption, even at lower cumulative doses. However, penetration is incomplete, indicating that additional genetic and non-genetic modifiers contribute to risk. A meta-analysis including four pediatric cohorts (817 children, 315 VIPN cases) reported no overall significant association between rs924607 TT and VIPN (OR = 1.99, 95% CI: 0.76–5.25, p = 0.16), but demonstrated a significant association during the continuation phase of therapy (OR = 2.28, 95% CI: 1.16–6.87, p = 0.02) (Zečkanović et al., 2020). Limitations included modest sample size, ethnic heterogeneity, and variability in neuropathy assessment. The ongoing St. Jude Total Therapy XVII trial (NCT03117751) is prospectively evaluating *CEP72* genotype-guided vincristine dosing to reduce VIPN incidence.

Genetic variation in drug transport pathways has also been investigated as a determinant of treatment-related toxicity. Polymorphisms in the *ABCB1* gene, which encodes the efflux transporter P-glycoprotein, have been associated with altered vincristine pharmacokinetics, neurotoxicity, and clinical outcomes in certain cohorts. In pediatric ALL, the variant rs4728709 may modulate VCR-induced grade I/II neurotoxicity (Nakagawa et al., 2023; Yuan et al., 2025; Ceppi et al., 2014). Specifically, carriers of the T allele exhibited a lower risk of toxicity (OR = 0.3; 95% CI: 0.1–0.9; p = 0.02), fewer neurotoxic episodes (p = 0.04), and tolerated higher VCR doses (p = 0.02). The C>T substitution thus appears to exert a protective effect against low-grade neurotoxicity (p = 0.01) (Nakagawa et al., 2023; Yuan et al., 2025; Ceppi et al., 2014). In contrast, rs1045642 and rs1128503, two variants in strong linkage disequilibrium (r = 0.725; p < 0.001), have been linked to altered transporter activity and reduced hepatic efflux of vincristine. Carriers of the rs1045642 AA genotype have also been reported to exhibit lower event-free survival, potentially reflecting increased systemic exposure to the drug (Yang et al., 2021).

However, several independent studies have failed to replicate consistent associations between common *ABCB1* variants and vincristine pharmacokinetics, toxicity, or prognosis (Hartman et al., 2010; Jamroziak et al., 2005). Functional investigations suggest that rarer missense variants, such as rs2229109 and rs2032582, may impair vincristine efflux, although their overall impact at the population level remains uncertain (Schaefer et al., 2006; Salama et al., 2006; Pozzi et al., 2020; Yang et al., 2021).

Additional loci identified through genome-wide and targeted association studies include genes involved in cytoskeletal organization and actin dynamics. The *ACTG1* variant rs1135989 has been significantly associated with high-grade VIPN and demonstrated predictive validity in an independent cohort (Abaji et al., 2018). Similarly, the *CAPG* promoter variant rs3770102 was associated with reduced risk of severe neuropathy, with AA genotype carriers showing lower toxicity incidence (OR 0.1; 95% CI 0.01–0.8; p = 0.009) and fewer neurotoxic episodes (Ceppi et al., 2014). These findings support a mechanistic role for actin remodeling in VIPN susceptibility, although further replication and functional validation are needed. Overall, associations between vincristine-related neurotoxicity and variants in *CYP3A5*, *CEP72*, *ABCB1*, *ACTG1* and *CAPG* are currently supported by moderate-level evidence (ClinPGx Level 3), derived mainly from candidate-gene studies, GWAS and functional investigations (Table 5).

**TABLE 5:** Genetic variants associated with vincristine toxicity.

Toxicity phenotype	Gene	Variant (rsID/HGVS nomenclature)	Study design/Genetic approach	Sample size (N), OR/HR (95% CI); p value	References	Allele frequency in reference populations
Peripheral neuropathy (VIPN)	*CYP3A5*	CYP3A5*3 NM_001291830.2 c.219–237A>G (rs776746)	Prospective cohort study	N = 1,204, OR = 1.02 (95% CI: 0.59–1.7)	Aplenc et al. (2003)	Global: 88% European: 93% African: 30% East Asian: 67%
			Retrospective cohort study/candidate gene association study	N = 508	Franca et al. (2017)	
			Retrospective cohort study/candidate gene association study	N = 339, p = 0.006	Ceppi et al. (2014)	
		CYP3A5*6 NM_001291830.2 c.624G>A (rs10264272)	Prospective cohort study	N = 1,204, OR = 0.79 (95% CI: 0.08–4.0)	Aplenc et al. (2003)	N/A
			Systematic review	​	Uittenboogaard et al. (2022)	
		CYP3A5*7 NM_001291830.2 c.1035dup (rs41303343)	Retrospective cohort study/Candidate gene association study	N = 239, OR = 1.33 (95% CI: 0.69–2.58)	McClain et al. (2018)	N/A
			Systematic review	​	Uittenboogaard et al. (2022)	
	*CEP72*	NC_000005.10: C>T (rs924607)	Genome-wide association study (GWAS)	N = 321, OR = 2.41 (95% IC: 1.70–3.49); p = 4.68 × 10^−8^	Diouf et al. (2015)	Global: 32.54% European: 41.47% African: 11.50% East Asian: 34.05%
			Meta-analysis of cohort studies/candidate gene association study	N = 315, OR = 2.28 (95% CI: 1.16–6.87); p = 0.02	Zečkanović et al. (2020)	
			Meta-analysis of cohort studies	N = 104, OR = 2.15 (95% CI: 1.35–3.43); p = 0.001	Klumpers et al. (2022)	
​	*ABCB1*	NM_001348946.2: c.-154–1146C>T (rs4728709)	Retrospective cohort study/candidate gene association study	N = 339, OR = 0.3 (95% CI: 0.1–0.9); p = 0.02	Ceppi et al. (2014)	Global: 7% European: 6% African: 35% East Asian: 16%
		NM_001348946.2: c.3435T>G/C/A (rs1045642)	Retrospective cohort study/candidate gene association study	N = 339, p = 0.03	Ceppi et al. (2014)	Global: 48% European: 47% African: 77% East Asian: 58%
			Prospective cohort study	N = 44, p = 0.98	Jamroziak et al. (2005)	
			Case-control study	N = 40, OR = 0.14 (95% CI: 0.02–0.86); p = 0.034	Stanulla et al. (2005)	
		NM_001348946.2: c.1236T>C (rs1128503)	Retrospective cohort study/candidate gene association study	N = 339, p = 0.1	Ceppi et al. (2014)	Global: 57% European: 57% African: 79% East Asian: 38%
		NM_001348946.2: c.2677T>G/A (rs2032582)	Retrospective cohort study/candidate gene association study	N = 339, p = 0.03	Ceppi et al. (2014)	Global: 56% European: 55% African: 87% East Asian: 51%
	*ACTG1*	NM_001614.5: c.930C>T (rs1135989)	Exome-wide association study (EWAS)	N = 237, p = 0.0001	Abaji et al. (2018)	Global: 30.05% European: 39.12% African: 18.83% East Asian: 0.52%
			Retrospective cohort study/candidate gene association study	N = 339, OR = 2.8 (95% CI: 1.3–6.3); p = 0.008	Ceppi et al. (2014)	
	*CAPG*	NM_001747.4: c.-14+3270C>T/A/g (rs3770102)	Retrospective cohort study/candidate gene association study	N = 339, OR = 0.1 (95% CI: 0.01–0.8); p = 0.009	Ceppi et al., 2014	Global: 35.48% European: 41.35% African: 33.14% East Asian: 9.36%

*CYP3A5*, Cytochrome P450, Subfamily IIIA, Polypeptide 5; *CEP72*, Centrosomal Protein, 72-KD; *ABCB1*, ATP-Binding Cassette, Subfamily B, Member 1; *ACTG1*, actin, Gamma-1; *CAPG*, capping protein, Gelsolin-Like; N/A, Not Available. Allele frequencies in reference populations were obtained from the Genome Aggregation Database (gnomAD).

### 3.6 Cyclophosphamide

Cyclophosphamide (CP), an oxazaphosphorine and bifunctional DNA-alkylating agent, remains a key component in the treatment of numerous pediatric and adult malignancies, including ALL. Its bioactivation occurs primarily in the liver through multiple cytochrome P450 isoenzymes: CYP2A6, CYP2B6, CYP2C8, CYP2C9, CYP2C19, CYP3A4 and CYP3A5, while the detoxification of its active metabolites is largely mediated by aldehyde dehydrogenases (ALDH1A1, ALDH3A1) and glutathione S-transferases (GSTA1, GSTM1, GSTP1, GSTT1). Functional polymorphisms within these enzymatic pathways have been associated with interindividual variability in cyclophosphamide-related toxicity across multiple disease settings and treatment regimens (Gervasini and Vagace, 2012). Following hepatic activation, cyclophosphamide yields two major metabolites: Phosphoramide mustard and Acrolein. Phosphoramide mustard, a nitrogen mustard derivative, represents the principal cytotoxic species by virtue of its ability to intercalate into DNA and induce cross-link formation, ultimately inhibiting replication and triggering cell death. In contrast, acrolein, generated during the conversion of 4-hydroxy-cyclophosphamide to phosphoramide mustard, is a highly reactive by-product responsible for most of cyclophosphamide-induced organ toxicities due to its potent alkylating and pro-inflammatory properties (Ibrahim et al., 2024).

#### 3.6.1 Mechanism of drug-induced toxicity

Cyclophosphamide (CP) is associated with a broad spectrum of adverse effects, including nephrotoxicity, hepatotoxicity, cardiotoxicity, neurotoxicity, genotoxicity, immunotoxicity, urotoxicity, and profound myelosuppression (Caglayan et al., 2018). A central mechanism underlying these toxicities is the excessive generation of reactive oxygen species (ROS), which drives oxidative stress and overwhelms endogenous antioxidant defenses. CP exposure increases the formation of superoxide anion (O_2_•–), hydroxyl radical (•OH) and hydrogen peroxide (H_2_O_2_), while concomitantly decreasing the activities of key antioxidant enzymes such as superoxide dismutase (SOD), catalase (CAT), glutathione peroxidase (GPx) and glutathione reductase (GR), ultimately promoting lipid peroxidation and oxidative injury to DNA, RNA, and proteins (Fahmy et al., 2016). The cytotoxic properties of CP are largely attributed to its metabolites, phosphoramide mustard and acrolein, generated through hepatic cytochrome P450–dependent bioactivation. Acrolein is a highly reactive aldehyde that amplifies ROS production, leading to lipid peroxidation, protein carbonylation, oxidative DNA damage, and activation of pro-inflammatory signaling pathways such as nuclear factor-κB (NF-κB), collectively culminating in hepatocyte injury and apoptosis, thereby contributing to CP-induced hepatotoxicity (Aladaileh et al., 2019). Similarly, CP-induced cardiotoxicity results from intertwined mechanisms involving oxidative and nitrosative stress, protein adduct formation, inflammatory activation within cardiomyocytes, disruption of calcium homeostasis, mitochondrial dysfunction, nuclear fragmentation, and the initiation of programmed cell death pathways (Iqubal et al., 2019). Together, these interconnected processes highlight oxidative stress, inflammation, and metabolite-mediated cellular damage as central drivers of cyclophosphamide-related toxicity (Figure 1).

#### 3.6.2 Genetic variants associated with cyclophosphamide toxicity

Multiple genes have been implicated in modulating cyclophosphamide induced toxicity (CTX). Among these, CYP2C19*2 (rs4244285) has been associated with an increased risk of gastrointestinal toxicity with a reported allele frequency of 33.09% in South Asian populations to 16.8% in the general population. Variants in detoxification enzymes also appear to influence toxicity: *GSTT1* complete deletion has been linked to hematological and other systemic toxicities, whereas *GSTP1* (rs1695) correlates specifically with gastrointestinal adverse effects (Yao et al., 2025). Transporter polymorphisms further contribute to interindividual variability. The *ABCC4* intronic variant rs9561778, present at a relatively uniform frequency of approximately 15% across populations (comparable to rs49561), has been associated with an elevated risk of gastrointestinal toxicity and neutropenia in patients receiving CTX-based regimens. Functional evidence suggests that rs9561778 may impair efflux of intracellular CTX or its active metabolites, resulting in increased intracellular accumulation and enhanced toxicity (Takahashi et al., 2022).

Collectively, these pharmacogenetic variants/*CYP2C19*, *GSTT1*, *GSTP1* rs1695 and *ABCC4* rs9561778 may provide a framework for predicting individual risk of cyclophosphamide-induced toxicities (Table 6). However, further validation in large, well-controlled cohorts and studies assessing multi-variant associations is required.

**TABLE 6:** Genetic variants associated with cyclophosphamide toxicity.

Toxicity phenotype	Gene	Variant (rsID/HGVS nomenclature)	Study design/Genetic approach	Sample size (N), OR/HR (95% CI); p value	References	Allele frequency in global reference populations
Gastrointestinal toxicity	*CYP2C19*	CYP2C19*2 NM_000769.4: c.681G>A (rs4244285)	Meta-analysis	N = 266, RR = 3.70 (95% IC: 1.60–8.55); p = 0.002	Yao et al. (2025)	Global: 16.80% European: 14.80% African: 17.74% East Asian: 31.23%
			Prospective cohort study/candidate gene association study	N = 85, HR = 0.08 (95% IC: 0.01–0.63); p = 0.05	Takahashi et al. (2022)	
Hematological toxicity	*GSTT1*	Gene deletion (null genotype)	Meta-analysis	N = 620, RR = 0.63 (95% IC: 0.42–0.96); p = 0.03	Yao et al. (2025)	N/A
Gastrointestinal toxicity	*GSTP1*	NM_000852.4: c.313A>G (rs1695)	Meta-analysis	N = 955, RR = 0.69 (95% IC: 0.52–0.92); p = 0.01	Yao et al. (2025)	Global: 35.99% European: 33.94% African: 44.27% East Asian: 18.04%
Gastrointestinal toxicity and neutropenia	*ABCC4*	NM_005845.5: c.3366+1243C>T (rs9561778)	Meta-analysis	N = 333, RR = 0.50 (95% IC: 0.28–0.88); p = 0.02	Yao et al. (2025)	Global: 17.47% European: 20.55% African: 14.46% East Asian: 28.15%

*CYP2C19*, Cytochrome P450 Family 2 Subfamily C Member 19; *GSTT1*, Glutathione S-Transferase Theta 1; *GSTP1*, Glutathione S-Transferase Pi 1; *ABCC4*, ATP, Binding Cassette Subfamily C Member 4; N/A, Not Available. No clinical guidelines or recommendations are currently applicable to any of the reported variants. Allele frequencies were obtained from the Genome Aggregation Database (gnomAD).

### 3.7 Glucocorticoids

Glucocorticoids, primarily dexamethasone and prednisone, constitute core components of multi-agent chemotherapy protocols for childhood ALL, being administered throughout induction, consolidation, and delayed intensification phases. Their antileukemic activity is mediated through binding to the glucocorticoid receptor (GR), encoded by *NR3C1*, which triggers transcriptional reprogramming characterized by repression of survival pathways and activation of pro-apoptotic signaling in lymphoid blasts, including BIM activation and inhibition of NF-κB and AP-1–dependent transcriptional programs (Pavlovic et al., 2019; Relling and Evans, 2015).

#### 3.7.1 Mechanisms of drug-induced toxicity

Despite their therapeutic efficacy, glucocorticoids are associated with substantial acute and long-term toxicities, including hypertension, hyperglycemia, immunosuppression, neuropsychiatric disturbances, osteoporosis and avascular necrosis (AVN) (Pavlovic et al., 2019). These adverse outcomes are strongly influenced by cumulative exposure and marked interindividual variability in pharmacokinetics and pharmacodynamics (Mei et al., 2015). In pediatric ALL, AVN represents one of the most severe complications, affecting approximately 10%–20% of patients, particularly adolescents exposed to prolonged high-dose dexamethasone regimens (Mattano et al., 2012).

#### 3.7.2 Genetic variants associated with glucocorticoids toxicity

Pharmacogenomic studies indicate that variability in glucocorticoid response and toxicity is, in part, genetically determined. Among candidate pharmacogenes, *NR3C1*, encoding the glucocorticoid receptor, represents a central molecular determinant of glucocorticoid sensitivity, signal transduction and downstream transcriptional responses (Pavlovic et al., 2019; Xue Y. et al. 2015; Xue L. et al., 2015).

While most investigations have focused on therapeutic response rather than toxicity, emerging data support a role for specific *NR3C1* variants in glucocorticoid-induced adverse effects. El-Fayoumi *et al.* demonstrated that the *N363S* polymorphism (*rs56149945)* is significantly associated with glucocorticoid-induced metabolic toxicity in pediatric ALL, with carriers exhibiting a markedly increased risk of glucose abnormalities compared to wild-type patients (El-Fayoumi et al., 2018). Complementary evidence from a pharmacogenetic retrospective cohort study conducted in a pediatric population further supports the contribution of *NR3C1* polymorphisms to glucocorticoid-related adverse effects during ALL therapy. This study, which included 49 children and adolescents with ALL and a control group of 46 pediatric patients with benign conditions, evaluated glucocorticoid-induced toxicities according to the NCI-CTCAE, version 4.0, and demonstrated associations between *NR3C1* variants (including N363S and BclI (rs41423247)) and the occurrence of metabolic and hepatic side effects (Kaymak Cihan et al., 2017). Beyond receptor biology, drug transport mechanisms play a critical role in modulating intracellular glucocorticoid exposure. *ABCB1*, encoding P-glycoprotein, an ATP-dependent efflux transporter, has emerged as a key pharmacogene influencing glucocorticoid pharmacodynamics. The *ABCB1 C3435T* (*rs1045642*) polymorphism has been linked to glucocorticoid-related toxicities, including bone marrow suppression and an increased risk of infectious complications (Marino et al., 2009; Pavlovic et al., 2019). This association was further supported by a pharmacogenetic population-based cohort study of 522 Danish pediatric patients, in which myelotoxicity was assessed using quantitative laboratory parameters (nadir hemoglobin, platelet and absolute neutrophil counts) rather than standardized grading systems (Gregers et al., 2015). Moreover, Gasic et al. found that the ABCB1 CGT haplotype was significantly associated with blast positivity on treatment day 8 (p = 0.018) (Gasic et al., 2018).

High-throughput genomic approaches have further expanded the understanding of glucocorticoid pharmacogenomics. Genome-wide association studies have identified polymorphisms in *ABCB1* associated with dexamethasone clearance and relapse risk, reinforcing the functional relevance of transporter-mediated pharmacokinetics in clinical outcomes. Additional loci, including variants near *GRIN3A* and within *ACP1*, have been associated with glucocorticoid-induced osteonecrosis in pediatric ALL, supporting a polygenic architecture underlying susceptibility to severe skeletal toxicity. Karol et al., in a genome-wide association study (GWAS) conducted in a predominantly pediatric cohort, identified an association between the SNP rs10989692, located near the glutamate receptor gene *GRIN3A*, and the risk of osteonecrosis (Table 7), with adverse events graded according to the NCI-CTCAE, version 3.0. (Karol et al., 2015; Pavlovic et al., 2019).

**TABLE 7:** Genetic variants associated with Glucocorticoids toxicity.

Toxicity phenotype	Gene	Variant (rsID/HGVS nomenclature)	Study design/Genetic approach	Sample size (OR/RR/HR, 95% CI; p value)	References	Allele frequency in reference populations
Hepatotoxicity	*NR3C1*	NM_001364182.1: c.1184+646C>G (rs41423247) (BclI)	Retrospective cohort study/candidate Gene association	N = 70, OR = 2.67 (95% CI: 0.53–7.33); p = 0.198	El-Fayoumi et al. (2018)	N/A
				N = 44 OR = 2.14 (95% CI: 0.45–10.1); p = 0.455	Kaymak Cihan et al. (2017)	
Bone marrow suppression and glucocorticoid-related toxicities	*ABCB1*	NM_001348946.2: c.3435T>C (rs1045642)	Retrospective Cohort study/candidate gene association	N = 122, p = 0.018	Gasic et al. (2018)	Global: 48% European: 47% African: 77%; East Asian: 58%
Osteonecrosis	*GRIN3A (near gene locus)*	NC_000009.12: G>A (rs10989692)	Retrospective cohort study/genome-Wide association study	N = 2,285, HR = 2.03 (95% CI: 1.55–2.66); p = 3.59 × 10^−7^	Karol et al. (2015)	Global: 11% European: 10% African: 43%; East Asian: 0.0%

*NR3C1,* Nuclear Receptor Subfamily 3 Group C Member 1*; ABCB1,* ATP-Binding Cassette Subfamily B Member 1*; GRIN3A,* Glutamate Ionotropic Receptor NMDA, Type Subunit 3A; N/A*,* Not Available. No clinical guidelines or recommendations are currently applicable to any of the reported variants. Allele frequencies were obtained from the Genome Aggregation Database (gnomAD).

Collectively, these data support a multifactorial model in which glucocorticoid toxicity and therapeutic response are governed by the integrated effects of receptor sensitivity (*NR3C1*), intracellular drug transport (*ABCB1*), systemic pharmacokinetics and downstream transcriptional and metabolic networks.

### 3.8 Tyrosine kinase inhibitors

Tyrosine kinase inhibitors (TKIs) have revolutionized the treatment of hematologic malignancies, including ALL, by selectively targeting dysregulated signaling pathways that drive leukemic cell proliferation and survival. In ALL, the Philadelphia chromosome–positive (Ph+) subtype and the related Ph-like entity are characterized by activation of tyrosine kinase signaling, most commonly through a constitutively active tyrosine kinase ABL1 kinase that represents a primary target of TKI therapy (Lee et al., 2011). While pharmacogenetic investigations in this context have mainly focused on genetic determinants of TKI efficacy, studies evaluating germline variants associated with TKI-related toxicity remain comparatively limited (Kong et al., 2012; Vivona et al., 2012). Despite their substantial therapeutic benefit, accumulating evidence indicates that TKIs are associated with cardiovascular adverse events that may compromise long-term outcomes.

#### 3.8.1 Mechanisms of TKI-induced cardiotoxicity and hypersensitivity

TKI-related cardiotoxicity arises from both on-target and off-target effects. Proposed mechanisms include mitochondrial dysfunction, endothelial injury, oxidative stress, dysregulation of calcium homeostasis, and inhibition of kinases essential for cardiomyocyte survival and vascular integrity. TKI therapy has been associated with QT interval prolongation, hypertension, left ventricular dysfunction, heart failure, and ischemic events, particularly for agents targeting ABL, VEGFR, and PDGFR pathways. Molecular and translational studies have begun to elucidate the biological basis of these toxicities, providing a rationale for exploring genetic susceptibility factors (Li et al., 2021).

#### 3.8.2 Genetic variants associated with TKI toxicity

Building on insights from studies of anthracycline-induced cardiotoxicity and underlying molecular mechanisms of cardiac injury, increasing attention has focused on tyrosine kinase inhibition as an important contributor to TKI-related cardiotoxicity (Force et al., 2007; Gupta et al., 2018; Gyöngyösi et al., 2020).

Yilan Li and colleagues conducted a genome-wide association study (GWAS) to prioritize single-nucleotide polymorphisms (SNPs) and functionally annotate genes associated with tyrosine kinase inhibitor (TKI)–induced heart failure (HF), thereby providing a foundation for gene variant association with TKI-related cardiotoxicity. Notably, multiple SNPs within the platelet-derived growth factor receptor alpha (*PDGFRA*) gene demonstrated strong regulatory potential, more likely to be functional in TKI-induced HF (Li et al., 2021).

Additional studies, mainly conducted in patients with chronic myeloid leukemia or gastrointestinal stromal tumors, have suggested that variant alleles such as CYP2D6*4 and *ABCG2* 421C>A affect imatinib clearance. Nevertheless, although these variants may be associated with altered TKI exposure and an increased incidence of adverse effects, their overall impact on pharmacokinetics appears to be limited (Gardner et al., 2006; Petain et al., 2008).

Although multiple studies have explored the impact of *ABCB1*, which encodes the efflux transporter P-glycoprotein, in response to tyrosine kinase inhibitor (TKI) therapy, data regarding its association with treatment-related toxicity remain scarce or absent. Previous findings indicate that *ABCB1* polymorphisms influence the sensitivity of *BCR::ABL1* expressing human leukemic cells to TKIs, particularly by affecting the response to imatinib. In contrast, these polymorphisms appear to have little or no effect on sensitivity to other TKIs, such as nilotinib, dasatinib, or ponatinib (Dessilly et al., 2016).

### 3.9 Innovative immunotherapies and emerging pharmacogenomic considerations

Innovative immunotherapeutic approaches for ALL, including CD19-directed chimeric antigen receptor T (CAR-T) cells and the bispecific T-cell engager blinatumomab, have markedly transformed the therapeutic landscape. However, these therapies are frequently associated with cytokine release syndrome (CRS), a potentially life-threatening complication resulting from excessive immune activation and elevated circulating cytokines, particularly interleukin-6 (IL-6) (Frey and Porter, 2016; Zhang et al., 2018). Standard management of CRS relies on supportive care and corticosteroids, whereas moderate to severe cases often require targeted modulation of IL-6–mediated inflammatory signaling. In this context, IL-6 receptor blockade with tocilizumab has demonstrated efficacy in mitigating CRS without compromising antitumor activity, establishing it as a cornerstone in the management of immunotherapy-related inflammatory toxicities in ALL.

Emerging evidence suggests that germline genetic variation within IL6R, which encodes the IL-6 receptor targeted by tocilizumab, may contribute to interindividual differences in treatment response and toxicity. Tocilizumab blocks both membrane-bound and soluble IL-6 receptors, and polymorphisms that influence receptor expression, structure, or downstream signaling could therefore modify the magnitude of IL-6 pathway inhibition. In a cohort of 88 patients with rheumatoid arthritis, six *IL6R* single-nucleotide polymorphisms (SNPs) were evaluated and several were significantly associated with distinct adverse-event profiles. The rs4845625 (T>C) variant was associated with an increased incidence of dyslipidemia among T-allele carriers, consistent with the role of IL-6 signaling in lipid metabolism. In contrast, rs11265618 (C>T), particularly the CC genotype, was associated with a higher frequency of hematologic toxicities, including neutropenia and thrombocytopenia. Similarly, individuals homozygous for the TT genotype at rs4329505 (T>C) exhibited a greater risk of hematologic adverse effects (Sainz et al., 2022). Collectively, these findings indicate that functional *IL6R* variants may modulate susceptibility to specific tocilizumab-related toxicities by altering the degree of IL-6 pathway suppression. Although these data derive primarily from non-oncologic populations, they provide a biologically plausible framework for pharmacogenomic investigations in patients receiving IL-6 receptor blockade for CRS in ALL (Sainz et al., 2022).

## 4 Discussion

The genetic determinants of chemotherapy-related toxicity in ALL (ALL) are highly heterogeneous. Among the numerous gene–toxicity associations reported for agents used in ALL treatment, variants in *TPMT* and *NUDT15* represent the most robust and consistently replicated predictors of mercaptopurine-induced myelosuppression. These genetic variants constitute clinically validated biomarkers and are recommended for routine genotyping by international consortia and regulatory or professional bodies, including the Clinical Pharmacogenetics Implementation Consortium (CPIC), the National Comprehensive Cancer Network (NCCN), and the U.S. Food and Drug Administration (FDA) through drug labeling (CPIC, 2024; NCCN, 2025; Food and Drug Administration (FDA), 2024). Notably, at St. Jude Children’s Research Hospital, prospective thiopurine dosing guided by *TPMT* genotype enabled dose reductions without compromising treatment efficacy, demonstrating the feasibility and clinical benefit of genotype-guided thiopurine therapy (Maillard et al., 2026). Importantly, recent CPIC updates have refined this framework by incorporating the combined effects of *TPMT* and *NUDT15* variation, highlighting both additive toxicity in dual intermediate metabolizers and the particularly high-risk profile associated with any poor metabolizer genotype. We also highlight the clinical recommendations of the Canadian Pharmacogenomics Network for Drug Safety (CPNDS) for pharmacogenomic testing of *RARG* rs2229774, *SLC28A3* rs7853758 and UGT1A6*4, which currently have the strongest and most consistent evidence for association with anthracycline-induced cardiotoxicity (Figure 1) (Aminkeng et al., 2016).

The ALLTogether study, a large European multicenter trial initiated in 2020, aims to improve pediatric/young adults- ALL overall survival and quality of survival, while also reducing unnecessary toxicity. By tailoring therapy across diverse risk groups, ALLTogether seeks to balance efficacy with reduced toxicity, underscoring the ongoing challenge of minimizing severe adverse events while maintaining optimal treatment outcomes in ALL-patients. The study also exemplifies an integrative precision medicine approach, combining tumor genomic profiling for risk stratification, therapeutic drug metabolite monitoring, and preemptive germline genotyping of *TPMT* and *NUDT15* to guide individualized dose adjustments (Heyman, 2025). Strategies such as treatment de-escalation for low-risk patients, together with monitoring and preemptive genotyping, represent complementary approaches to minimize toxicity while maintaining therapeutic efficacy in ALL therapy (Guo et al., 2022).

Preemptive genotyping is still in its early stages in the clinical routine. Apart from mercaptopurine-related genetic variations, which have been implemented in several specialized centers, numerous pharmacogenetic associations with ALL drug-related toxicities remain investigational. Further research is needed to establish robust links between genetic variation and drug toxicity that can be translated into clinically actionable pharmacogenomic biomarkers. These include pharmacogenetic associations related to glucocorticoids, L-asparaginase, vincristine, anthracyclines, methotrexate, cyclophosphamide, tyrosine kinase inhibitors, and immunomodulatory agents, as outlined above.

When reviewing genetic variants associated with ALL treatment-related toxicities, it is important to highlight the recurrent implication of common metabolic and transport pathways across multiple drug–toxicity associations, while others are specific to certain drugs (Figure 1). Genetic modulation of metabolic and transport pathways may underlie toxicities shared across multiple agents included in ALL protocols and may also contribute to cumulative or synergistic toxicities observed with multi-agent regimens commonly used in ALL treatment.

A Common metabolic and transport pathways across multiple drug–toxicity associations is the ATP-binding cassette (ABC) transporters, *ABCB*1 (rs1045642, rs4728709), *ABCC1* (rs246240), *ABCC2* (rs3740065), *ABCC4* (rs7317112, rs9561778), have been associated with toxicity related to glucocorticoids, vincristine, methotrexate and cyclophosphamide, agents that are frequently co-administered in ALL treatment protocols. Beyond ABC transporters, polymorphisms in solute carriers have been implicated in ALL-related toxicities, particularly gastrointestinal toxicity and cardiotoxicity; Variants in *SLCO1B*1 (rs4149081, rs11045879) and *SLC28A3* (rs7853758, rs885004), have been associated with toxicities induced by methotrexate and anthracyclines. Polymorphisms in glutathione S-transferases detoxification enzymes, GSTA1, GSTM1, have been associated with susceptibility to hematological and gastrointestinal toxicities, particularly in the context of cyclophosphamide treatment. Drug-metabolizing Cytochrome P450 (CYP) superfamily enzymes not only take part in the transformation of most of the clinically used drugs (90%) but also toxins and carcinogens (Li and Bluth, 2011). They may represent another major determinant of interindividual variability in chemotherapy-related toxicities. For instance, CYP2C19*2 (rs4244285) has been associated with an increased risk of gastrointestinal toxicity associated with Cyclophosphamide, CYP2D6*4 potentially associated with ITK-Cardiotoxicity, while polymorphisms in *CYP3A5* (rs776746, rs10264272, rs41303343), have been linked to vincristine-induced peripheral neuropathy (VIPN).

Beyond the convergence of genetic associations within drug metabolism pathways, notable associations have also been reported for transcriptional regulators. For example, the *RARG* variant (rs2229774) has been associated with anthracycline-induced cardiotoxicity, potentially through altered regulation of RARG target genes. Similarly, *CNOT3* variants (rs73062673) have been implicated in asparaginase hypersensitivity; CNOT3 is a core component of the CCR4–NOT complex, which plays a crucial role in post-transcriptional regulation of gene expression. In addition to germline variant–based approaches, including candidate-gene studies and genome-wide association studies (GWAS), transcriptomic strategies may further refine the prediction of drug toxicity and therapeutic response (Maillard et al., 2026). For example, TPMT expression levels are modulated by promoter variants, particularly variable number tandem repeats (VNTRs), which influence transcript abundance and contribute to interindividual variability in thiopurine tolerance (Kotur et al., 2012; Zukic et al., 2010). While numerous transcriptomic studies in ALL have focused on expression signatures defining and classifying ALL-genetic subtypes, investigations specifically addressing *in vivo* transcriptomic toxicity profiles remain limited (Lilljebjörn et al., 2022; Pavlovic et al., 2019; Tran et al., 2022; Walter et al., 2021).

Zaza and colleagues used microarray gene expression profiling to analyze approximately 9,600 genes in ALL cells obtained at diagnosis. They identified 60 gene probes significantly associated with intracellular thioguanine nucleotide (TGN) accumulation in patients treated with mercaptopurine (MP) alone and 75 probes in patients receiving combination therapy with methotrexate (MTX) and MP. Genes associated with TGN accumulation after MP monotherapy included those encoding MP metabolic enzymes and transporters, such as *SLC29A1*, xanthine oxidase (*XDH*), and aldehyde dehydrogenase one family member A2 (*ALDH1A2*), highlighting the influence of both transport and metabolic pathways on thiopurine pharmacokinetics (Zaza et al., 2005).

MicroRNAs (miRNAs), which regulate gene expression and may modulate treatment-related toxicities in ALL, have been investigated in only a few studies. For example, the A allele of rs12402181 in the seed region of miR-3117-3p, which could alter binding to *ABCC1* and *RALBP1*, and the C allele of rs7896283 in the precursor sequence of miR-4481, have been significantly associated with vincristine-induced neurotoxicity. These findings suggest that miRNA-mediated regulation of pharmacogenes may contribute to interindividual variability in chemotherapy toxicity (Gutierrez-Camino et al., 2018; López-López et al., 2014; Doughan et al., 2023). Integrating multiple layers of molecular data, germline variants, gene expression profiles and miRNA regulation, through a multi-omics approach may strengthen the associations with drug toxicity and improve the prediction of adverse events in ALL therapy.

Several challenges hinder the development and clinical implementation of predictive pharmacogenomic biomarkers in ALL treatments. The robustness of many reported gene–drug associations still requires validation in larger, multiethnic cohorts. Notably, *NUDT15*, now recognized as a major determinant of thiopurine toxicity, was identified more than 20 years after the characterization of *TPMT* variants, highlighting the importance of studying pharmacogenomics in diverse populations, where allele frequencies may differ markedly (Shriver et al., 2024). Particularly within the highly polymorphic HLA region, which varies markedly across populations, studies in diverse ancestral groups are of particular importance. For example, HLA-B*75 serotype alleles have been associated with carbamazepine-induced severe and life-threatening cutaneous adverse reactions in epilepsy treatment, predominantly in Asian populations, while these associations have been difficult to replicate in other populations (Masmoudi et al., 2022; Chung et al., 2004). This underscores the importance of ancestry-specific studies in HLA pharmacogenetics in ALL treatment, particularly for asparaginase-related hypersensitivity reactions.

Patients often receive combinations of agents with overlapping toxicity profiles, such as hepatotoxicity and myelosuppression, which complicates attribution of adverse effects to a single drug (Maamari et al., 2020). Some studies have investigated gene–variant associations with toxicity during specific therapy phases rather than with a single drug. While this review maintains a drug-specific structure, we have cited gene–variant associations reported for multi-agent induction or consolidation phases when possible. Phenotype harmonization and standardized study endpoints in multicenter studies could further advance the establishment of reliable toxicity–genetic associations.

With the advent of pan-genomic technologies, targeted gene panel approaches have been increasingly investigated to prevent drug-related toxicities. In a multicenter implementation study, the Preemptive Pharmacogenomic Testing for Preventing Adverse Drug Reactions (PREPARE) trial evaluated a 12-gene pharmacogenetic panel and demonstrated a reduced incidence of clinically relevant adverse drug reactions, as well as feasibility across diverse European healthcare systems and settings. The panel included *CYP2B6*, *CYP2C9*, *CYP2C19*, *CYP2D6*, *CYP3A5*, *DPYD*, *F5*, *HLA-B*, *SLCO1B1*, *TPMT*, *UGT1A1* and *VKORC1*; Patients with actionable variants were managed according to international pharmacogenetic guidelines (Swen et al., 2023). Specifically in pediatric populations, the PG4KDS program at St. Jude Children’s Research Hospital evaluated the preemptive utility and feasibility of pharmacogenetics. Using the Affymetrix Drug Metabolizing Enzymes and Transporters Plus array, variants in 230 genes were assessed, and four gene tests (*TPMT*, *CYP2D6*, *SLCO1B1*, and *CYP2C19*) were integrated into the electronic health record (EHR) for clinical implementation. These tests were coupled to 12 high-risk drugs, providing a framework for personalized therapy and reduction of drug-related toxicities (Hoffman et al., 2014).

This biological convergence across multiple drug–toxicity associations supports the use of pathway-based and multigene panel approaches and further motivates studies to investigate the combined effects of multiple variants across related genes. Beyond multigene panels, polygenic risk scores (PRS), which integrate the cumulative impact of numerous variants across relevant genes, have the potential to enhance the predictive power of genetic testing for guiding and adjusting treatment (Bienfait et al., 2022; Hoffman et al., 2014). Studies evaluating hundreds to thousands of variants or gene expression profiles may enable the generation of a single numerical score that estimates an individual’s genetic predisposition and predicts their risk of drug toxicity.

It is important to underscore the need for clinical decision support tools and the continuous education of pharmacists and physicians to facilitate the implementation of pharmacogenomics in routine healthcare settings (Peruzzi et al., 2025). In addition, research efforts should be sustained from the perspectives of health economics and implementation science. New study designs and modeling approaches are required to evaluate the cost-effectiveness of multigene testing, particularly given its evolving and increasingly widespread use, as well as the complexity of interpreting multiple test outputs. Multigene panels can simultaneously assess not only diverse pharmacogenes but also somatic leukemia variants for leukemia risk stratification within a single assay, offering significant advantages for integrated precision medicine approaches (Gavan et al., 2018; Geyer et al., 2025). The robustness of a gene variant or polygenic risk score (PRS) association with drug toxicity is critical for its clinical implementation, as reflected in its positive and negative predictive values (PPV and NPV) for preventing adverse events. These metrics provide essential input parameters for cost-effectiveness and budget impact analyses, helping to determine the clinical and economic value of implementing pharmacogenomic testing (Hughes et al., 2004; Fuerte et al., 2025; TPMT genotyping before mercaptopurine cost effective,” 2006).

To expand the potential of preemptive genotyping for preventing toxicities in leukemia care and ensure its implementation in clinical practice, future efforts should focus on validating emerging biomarkers and developing integrative predictive strategies. This includes investigating pharmacogenetic markers in large, multiethnic cohorts, exploring polygenic risk scores, and applying artificial intelligence–driven models to enhance toxicity prediction and support precision medicine in pediatric ALL. Importantly, these efforts should be inclusive of low- and middle-income countries to avoid further widening the existing disparities in survival outcomes.
